# Physicochemical characteristics of structurally determined metabolite-protein and drug-protein binding events with respect to binding specificity

**DOI:** 10.3389/fmolb.2015.00051

**Published:** 2015-09-15

**Authors:** Paula Korkuć, Dirk Walther

**Affiliations:** Max Planck Institute for Molecular Plant PhysiologyPotsdam-Golm, Germany

**Keywords:** metabolites, drugs, protein binding, promiscuity, physicochemical properties, partial least squares (PLS), pathway enrichment analysis

## Abstract

To better understand and ultimately predict both the metabolic activities as well as the signaling functions of metabolites, a detailed understanding of the physical interactions of metabolites with proteins is highly desirable. Focusing in particular on protein binding specificity vs. promiscuity, we performed a comprehensive analysis of the physicochemical properties of compound-protein binding events as reported in the Protein Data Bank (PDB). We compared the molecular and structural characteristics obtained for metabolites to those of the well-studied interactions of drug compounds with proteins. Promiscuously binding metabolites and drugs are characterized by low molecular weight and high structural flexibility. Unlike reported for drug compounds, low rather than high hydrophobicity appears associated, albeit weakly, with promiscuous binding for the metabolite set investigated in this study. Across several physicochemical properties, drug compounds exhibit characteristic binding propensities that are distinguishable from those associated with metabolites. Prediction of target diversity and compound promiscuity using physicochemical properties was possible at modest accuracy levels only, but was consistently better for drugs than for metabolites. Compound properties capturing structural flexibility and hydrogen-bond formation descriptors proved most informative in PLS-based prediction models. With regard to diversity of enzymatic activities of the respective metabolite target enzymes, the metabolites benzylsuccinate, hypoxanthine, trimethylamine N-oxide, oleoylglycerol, and resorcinol showed very narrow process involvement, while glycine, imidazole, tryptophan, succinate, and glutathione were identified to possess broad enzymatic reaction scopes. Promiscuous metabolites were found to mainly serve as general energy currency compounds, but were identified to also be involved in signaling processes and to appear in diverse organismal systems (digestive and nervous system) suggesting specific molecular and physiological roles of promiscuous metabolites.

## Introduction

Metabolic conversion processes require a close physical contact between metabolite substrates and their cognate protein enzymes acting on them. Substrate specificity and the kinetics of the substrate-enzyme encounter are encoded by the details of the molecular recognition process, which are determined by the physicochemical properties of both interaction partners (Volkamer et al., 2013).

Beyond being involved in enzymatic conversion processes, evidence is accumulating that metabolites can serve signaling functions as well (Yang et al., 2012; Li et al., 2013). Early findings uncovered the metabolite-binding mediated allosteric effects of metabolites on enzymatic activity (Monod et al., 1965). Specific signaling roles of metabolites have furthermore been established in a broad array of processes ranging from riboswitches in bacteria [i.e., interaction with RNAs (Mandal and Breaker, 2004)] to the regulation of flowering in plants (Wahl et al., 2013), and to hormonal regulations in human (Aranda and Pascual, 2001). To what extend metabolites in general exert a signaling role remains a central research question.

As putative signaling roles of metabolites can be assumed to be mediated by physical interactions with other molecules (proteins, DNA, RNA), understanding the interactions of metabolites with proteins, in particular, may provide clues for potential signaling activities. Here, gauging target specificity based on physicochemical properties is of central interest. Metabolites with a broader protein target range may more likely also fulfill signaling functions in addition to their role as substrate in biochemical reaction. In a seminal experimental study, the potential of interactions of metabolites with proteins implicated in signaling (kinases) has been demonstrated in yeast (Li et al., 2010). Binding promiscuity may also be associated with unspecific metabolic conversions or cross-reactivities, in which enzymes process metabolites other than their canonical substrates. This “accidental” reactivity has also been discussed as a mode of metabolic network evolution (Carbonell et al., 2011). Thus, approaching promiscuity from the perspective of protein binding sites rather than regarding promiscuity a property of compounds alone may allow predicting non-canonical enzymatic reaction and may thus contribute to furthering our understanding of metabolic reactions and the resulting set of naturally occurring metabolic compounds in biological systems. In fact, results from computational docking studies on metabolite-enzyme interactions in *E.coli* suggest that promiscuity may indeed originate from both substrates and enzymes properties (Macchiarulo et al., 2004). As a long term goal, the prediction of enzymatic reactions based on the structure of enzymes and compound substrate alone may also prove instrumental for the annotation of recorded mass-spectra associated with detected metabolites in biological samples, whose identity presently remains unknown (Anari et al., 2004). Furthermore, understanding metabolite-protein binding events may provide clues for the mechanisms that underlie observed correlated metabolomic and transcriptomic changes in cellular systems exposed to stress conditions (Bradley et al., 2009; Walther et al., 2010). If it proves possible to correctly predict target proteins of metabolites, the signaling cascade leading to transcriptional changes may become decipherable.

Thus, a detailed survey and characterization of experimentally observed and structurally resolved metabolite-enzyme binding events as reported in the Protein Data Bank (PDB) appears worthwhile and motivated this study. Toward achieving the more general goal of understanding the physicochemical determinants of compound-protein binding events leading ultimately to the ability to predict metabolite-protein binding events, the inclusion of all protein binding events—including metabolites bound to non-catalytic sites—as well as considering compounds other than metabolites alone will allow broadening the available dataset and may uncover general principles of compound-protein encounters.

The study of compound-protein interactions has been at the core of drug development programs for decades. As high specificity of protein target binding is considered desirable for the therapeutic success, the factors influencing binding specificity of drug compounds have been investigated intensively, and their continued study remains a central research objective in both academia and pharmaceutical industry. As it may cause adverse side effects, promiscuous binding of drugs to many off-target proteins is of particular concern (Lounkine et al., 2012; Hu and Bajorath, 2013; Rudmann, 2013; Hu et al., 2014). Experimental as well as computational studies have generated a wealth of knowledge on the rules that govern the association of physicochemical properties of drug compounds and their target protein spectrum (Tarcsay and Keserű, 2013). On the other hand, unexpected binding to off-targets may also help to position established drugs for novel medicinal indications (for review of positive and negative effects of promiscuity see Peters, 2013). To probe for promiscuity and other ADME (absorption, distribution, metabolism, and excretion) properties, appropriate representative protein panels have been established, with which compound promiscuity can be assayed experimentally (Krejsa et al., 2003). Because detailed computational all-against-all docking studies proved prohibitive (for lack of structural information or limiting computational power), such experimental binding surveys have been analyzed to establish general rules that associate physicochemical properties of compounds with binding promiscuity of drugs. For example, it was found that lipophilicity (logP) and basic character (pK_a_) appear positively correlated with promiscuous binding behavior (Tarcsay and Keserű, 2013).

In this study, we performed a systematic analysis of metabolite-protein interactions and compared them with the characteristics of drug-protein binding events. We based our analysis on observed interactions of small compounds with proteins in the PDB as has been done for drugs (Haupt et al., 2013) and drug-like compounds (Sturm et al., 2012) before. Here, we extended the analysis to include naturally occurring metabolites and to reveal possible similarities and differences between the two compound sets with regard to protein binding behavior thereby examining the transferability of approaches, algorithmic concepts, and physiochemical principles from the rich drug development field to the realm of metabolomics. A large number of physicochemical properties was profiled and their influence on the binding characteristics investigated. In particular, we assessed the degree of specificity/promiscuity of compounds with respect to their underlying chemical structure. We studied promiscuity from the perspective of compound-based as well as protein-target-based properties applying both descriptive and predictive statistical approaches. A plethora of studies has been devoted to the computational analysis and prediction of compound-protein interactions. However, given their pharmacological relevance, such studies have mainly focused on drug-protein interactions (Carbonell and Faulon, 2010; Yabuuchi et al., 2011; Yu and Wild, 2012; Haupt et al., 2013; Ding et al., 2014). Computational studies on metabolite-protein contacts were mostly concerned with predicting substrate-enzyme interactions (Macchiarulo et al., 2004; Carbonell and Faulon, 2010) and specific metabolites (Stockwell and Thornton, 2006; Kahraman et al., 2010) rather than to also investigate generic binding modes of metabolites. The present study presents a broader, integrative survey with the aim to elucidate common as well as set-specific characteristics of compound-protein binding events and to possibly uncover specific physicochemical compound properties that render metabolites candidates to serve as signals.

## Materials and methods

### Compound-protein target datasets

#### Metabolites

Initial metabolite sets were obtained from (i) the Chemical Entities of Biological Interest database (Degtyarenko et al., 2008) (ChEBI, version 2014/07/07) comprising 5771 metabolite structures classified under ChEBI ID 25212 ontology term “metabolite,” (ii) the Kyoto Encyclopedia of Genes and Genomes (Kanehisa and Goto, 2000) (KEGG, version 2014/12/07, 15,519 compounds), (iii) the Human Metabolome Database (Wishart et al., 2007) (HMDB, version 3.6, 2014/04/13, 41,498 compounds), and (iv) the MetaCyc database (Caspi et al., 2014) (version 18.0, 2014/06/18, 12,713 compounds). KEGG compounds structures were downloaded using the KEGG API (http://www.kegg.jp/kegg/docs/keggapi.html). Metabolites from KEGG and MetaCyc were converted from MDL Molfile to SDF format using OpenBabel (O'Boyle et al., 2011). The union of all four sets was shortlisted for those metabolites contained also in the Protein Data Bank (PDB).

#### Drugs

Chemical structures of all non-nutraceutical small molecule drugs (approved and experimental) were downloaded as structure-data files (SDF) from the DrugBank database (Wishart et al., 2006) (version 4.1, 2014/09/08) comprising a total of 6858 drug molecules.

#### Protein targets and co-crystallized compounds

To generate the protein target set associated with all compounds, all available protein structures with at least one co-crystallized, non-covalently bound compound and a X-ray crystallographic resolution of 2Å or better were downloaded from the Protein Data Bank (Berman et al., 2000) (PDB, version 2014/07/31). In case of protein structures with multiple amino acid chains, every chain was considered separately as potential compound targets. Targets bound only by very small (<30 Da), very large compounds (>1000 Da), common ions (e.g., Na^+^, Cl^−^, SO${}_{4}^{-}$), solvents (e.g., water, MES, DMSO, 2-mercaptanol, glycerol), chemical fragments or clusters were removed from the dataset (Powers et al., 2006).

#### Compound binding pockets

Compound binding pockets were defined as compound-protein interaction sites with at least three separate target protein amino acid residues engaging in close physical contacts with a given compound. Contacts were defined as any heavy protein atom to any heavy compound atom within a distance of 5Å.

Redundant or highly similar binding pockets resulting from multiple binding events of the same compound to a particular target protein were eliminated. All binding pockets of the same compound found on the same protein were clustered hierarchically (complete linkage) with regard to their amino acid composition using Bray-Curtis dissimilarity, *d*_*BC*_, calculated as:

(1) $$d_{BC}=\frac{\sum_{i\;=\;1}^{n}\left|a_{i}-b_{i}\right|}{\sum_{i\;=\;1}^{n}\left(a_{i}+b_{i}\right)},$$

where *a*_*i*_ and *b*_*i*_ represent the counts of amino acid residues *i* = 1, …, *n* (*n* = 20) of two individual pockets. The clustering cut-off value was set to 0.3 keeping one representative binding pocket of each cluster.

To remove redundancy between protein targets, the set of all protein targets associated with each compound was clustered according to 30% sequence similarity cutoff using NCBI Blastclust (Dondoshansky and Wolf, 2002) keeping one representative of each cluster (parameters: score coverage threshold = 0.3, length coverage threshold = 0.95, with required coverage on both neighbors set to FALSE). As a result, each compound was associated to a non-redundant and non-homologous target pocket dataset.

The chemical structures of those 7385 compounds, for which a target protein was identified in the PDpB, were downloaded as ideal CCD (Chemical Compound Dictionary) coordinates (http://www.wwpdb.org/ccd.html).

#### Compound promiscuity

Compounds bound to three or more non-redundant target pockets were defined “promiscuous,” all others “selective.”

### Compound classification and property calculation

Molecular weights and SMILES strings (“Simplified Molecular Input Line Entry Specification”) of all compound structures were calculated using the Instant JChem software (version 14.7.7.0, ChemAxon, http://www.chemaxon.com). Very small or large compounds (molecular weight <30 Da or >1000 Da), variable compound structures comprising R-groups and compounds without computable SMILES were not considered for further analysis. The chemical development kit (CDK) extended fingerprints from the *rcdk* R-package (Guha, 2007) was used for similarity analysis of compound structures. Drugs or metabolites were mapped to PDB compounds requiring identical molecular weights (at ± 1 Da tolerance) and identical fingerprint (Tanimoto distance, T, T > 0.95; 91% of all compounds mapped with *T* = 1.0). PDB compounds assigned to both drug and metabolite compounds were labeled as “overlapping compounds.”

Physicochemical properties of those the compound class considered here (drugs, metabolites, and overlapping compounds) were calculated by using Instant JChem and KNIME (Berthold et al., 2008) (version 2.9.4) (The list of all computed properties is provided in Supplementary Figure 1). Properties based on actual 3D-structures were based on the ideal Chemical Compound Dictionary (CCD) compound coordinates (http://www.wwpdb.org/ccd.html).

### Compound-promiscuity propensity ratio calculation

Physicochemical properties preferentially associated with either promiscuous or selective compounds (Table 1B) were judged based on propensity values, *P*, calculated for each property type *t* and compound class *c* as:

(2) $$P_{i}^{t,c}=\frac{f_{i}}{g_{i}}=\frac{q_{i}/\sum_{i\;=\;1}^{n}q_{i}}{s_{i}/\sum_{i\;=\;1}^{n}s_{i}},$$

where *q* is the frequency of promiscuous compounds within a property range interval *i* divided by the sum of promiscuous compound counts over all intervals *i* = 1, …, *n*. This term is divided by the relative frequency of selective compounds *s* within interval *i* divided by the sum of all compound counts over the intervals *i* = 1, …, *n*. The intervals were chosen to ensure that all intervals contain nearly the same compound count. Standard errors, *se*, of the obtained propensities were calculated as defined in Levitt (1978) with:

(3) $$se_{i}=\frac{1}{g_{i}}\sqrt{\frac{f_{i}(1-f_{i})}{\sum_{i\;=\;1}^{n}q_{i}}}$$

**Table 1:** **Overview of the drug and metabolite compound sets used in this study**.

**(A)**					
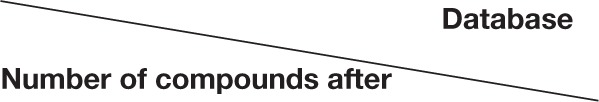	**Drugs**	**Metabolites**			


	**Drugbank**	**ChEBI**	**KEGG**	**HMDB**	**MetaCyc**
Download	6858	5771	15,519	41,498	12,713
Filtering	6566	5405	15,031	34,785	10,250
Assignment to PDB compounds	2227	217	1304	1100	1013
**(B)**					
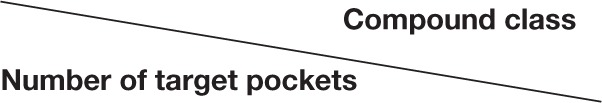	**Drugs**	**Metabolites**	**Overlapping compounds**	**All compounds**	
> = 1	1226 (3271)	659 (2600)	1001 (6551)	2886 (12,422)	
> = 2	250 (2295)	226 (2167)	562 (6112)	1038 (10,574)	
> = 3	114 (2023)	129 (1973)	395 (5778)	638 (9774)	
> = 4	65 (1876)	85 (1841)	298 (5487)	448 (9204)	
> = 5	44 (1792)	56 (1725)	232 (5223)	332 (8740)	

*(A) Number of drug and metabolite structures downloaded from DrugBank, ChEBI, KEGG, HMDB, and MetaCyc, filtered according to molecular weights and SMILES computability, and assigned to PDB compounds. (B) Number of PDB compounds categorized as drugs, metabolites or overlapping compounds that are bound to at least 1, 2, etc. non-redundant protein target pockets. The numbers of interacting target pockets are listed in parentheses*.

Propensity values were log_10_-transformed to produce symmetrical distributions.

### Amino acid residue compositional propensities of protein binding pockets

Compound binding pocket amino acid composition propensities were calculated using Equation (2), followed by log_10_-transformation and with *q*_*i*_ representing the number of amino acid residues of type *i* = 1, …, 20 in binding pockets and *s*_*i*_ the number of amino acid residues *i* = 1, …, *n* in non-binding site parts of proteins.

### Enzyme classification entropy and pocket variability analysis

The degree of target set variability associated with each promiscuous compound was characterized by two measures, the entropy of EC numbers of target proteins and the variability of amino acid composition of binding pockets.

#### EC entropy

For every compound, the number of target-protein-associated EC numbers was counted. The six top-levels of the EC number classifications were used only, where “EC 1” represents oxidoreductases, “EC 2” transferases, “EC 3” hydrolases, “EC 4” lyases, “EC 5” isomerases, “EC 6” ligases (http://www.chem.qmul.ac.uk/iubmb/enzyme/). The label “None” was introduced for target proteins without EC number assignment. The resulting counts were normalized to the total number of elements in every EC class and the total number of EC assignments within each compound's target set. The entropy *H* was computed from these probabilities *p*_*i*_ of the EC classes *i* = 1,.,n (*n* = 7) for each compound as:

(4) $$H=-\sum_{i\;=\;1}^{n}p_{i}ln(p_{i}).$$

For compounds with highly diverse EC classification numbers, the entropy tends toward the maximum value of log_2_ (n), and toward 0 for compounds with only few EC classes. Note that for the entropy calculation, the number of different targets was based on protein target counts, not binding pockets leaving 545 promiscuous compounds for analysis.

#### Protein binding pocket variability, PV

The variability of binding pockets associated with a given compound was assessed based on the variation of amino acid composition of binding pockets across all binding events and termed “pocket variability.” The pocket variability, *PV*, was calculated for each compound's target pocket set as:

(5) $$PV=\sum_{i\;=\;1}^{n}\frac{\sigma_{i}^{2}}{\mu_{i}},$$

where $\sigma_{i}^{2}$ represents the variance and μ_*i*_ the mean of the count of amino acid residue *i* = 1, …, *n* (*n* = number of different amino acid residue types involved in binding) within the target pocket set associated with a given compound. Six hundred and thirty-eight compounds with at least three non-redundant target pockets were included in these calculations (see Table 1B). Please note that *PV* is independent of the size of the compound and associated number of amino acid residues types involved in binding.

### Binding mode prediction models

Partial least squares regression models (PLSR) were built using the *pls* R-package (Mevik and Wehrens, 2007) for the target variables EC entropy, pocket variability, and number of compound target pockets (log10) for all compounds jointly and separately for the three compound classes drugs, metabolites, and overlapping compounds. The set of physicochemical properties was used as predictor variables. The optimal number of principal components was selected using the component number with the lowest root mean squared error of prediction (RMSEP) of the initially maximally allowed 10 components.

Support Vector Machines were created using the *kernlab* R-package (Karatzoglou et al., 2004). The variables were scaled and a 5-fold cross-validation was performed on the training data to assess the quality of the model.

Classification and regression trees were created using the *rpart* and *partykit* R-packages (Therneau and Atkinson, 1997; Hothorn and Zeileis, 2012), where each tree was pruned according to the lowest cross-validated prediction error within a range of 3–10 tree splits.

### Metabolite pathway, process, and organismal systems enrichment analysis

Pathway mappings used in the enrichment analysis were obtained from KEGG (http://www.genome.jp/kegg/pathway.html, 2014/08/12). In total, 323 of the 659 available metabolite compound structures (see Table 1B) were also present in KEGG pathway maps. Pathway maps were partitioned into seven generic classes, of which only “Metabolism,” “Environmental Information Processing,” and “Organismal systems” comprised a sufficient number (> = 20) of unique metabolic compounds, and thus were used for analysis. The enrichment analysis was performed using both the collective map terms, which, for instance, sum up all carbohydrate pathways in the “Metabolism” class or all membrane transport systems in the “Environmental information processing” class, and the detailed pathway names, e.g., glycolysis, citrate cycle, and pentose phosphate pathway, which are part of the collective map of “Carbohydrate metabolism” in “Metabolism” class. The maps of “Metabolism,” “Environmental Information Processing,” and “Organismal Systems” comprised 14, 4, 10 collective terms and 165, 24, 64 detailed terms, respectively. The set of compounds used in this study was mapped to 12, 4, and 8 collective terms and 125, 16, and 23 for detailed terms.

Enrichment or depletion of specific pathway annotations found in a particular compound set relative to another was tested by applying Fisher's exact test (Fisher, 1929). The resulting *p*-values were corrected for multiple testing applying the Benjamini-Hochberg procedure (Benjamini and Hochberg, 1995).

## Results

### Compound-protein target dataset

For the characterization of physical and structurally resolved interactions of metabolites with proteins and comparing them with drug-protein binding events, first a suitable dataset comprising compounds and their target proteins had to be assembled. We downloaded all available protein-compound complex structures from the Protein Data Bank (PDB) with a crystallographic resolution of 2Å or better and removed all binding events involving particularly small or large compounds, common ions, solvents, chemical clusters, or fragments. We rendered the protein target set non-redundant by clustering them according to a sequence identity of 30% using NCBI Blastclust to get for each of those PDB-derived 7385 compounds a non-homologous and non-redundant target set (see Materials and Methods).

We treated PDB compounds as drugs or metabolites based their match to compounds contained in DrugBank or metabolite databases (ChEBI, KEGG, HMDB, and MetaCyc), respectively. Matches were established based on near identical molecular weights and chemical fingerprints. PDB compounds that could be assigned to both drugs and metabolites were labeled as “overlapping compounds” (see Materials and Methods). We considered a compound promiscuous, if it binds to three or more target protein binding pockets, whereas compounds with one or two binding events were classified as “selective.” The final dataset comprised 2886 PDB compounds with at least one non-redundant target pocket and 1226 of them classified as drugs, 659 as metabolites, and 1001 as both and thus are termed “overlapping compounds” (Table 1A). 638 compounds (22%) of those PDB compounds are promiscuous. They include 114 drugs, 129 metabolites, and 395 overlapping compounds, which altogether interact with 9774 target pockets (Table 1B). As already evident from the statistic, drug compounds are much more selective, with 9.3% qualifying as promiscuous, than metabolites (19.5% promiscuous).

### Physicochemical properties of metabolites and drugs bound to proteins

**Figure 1:**
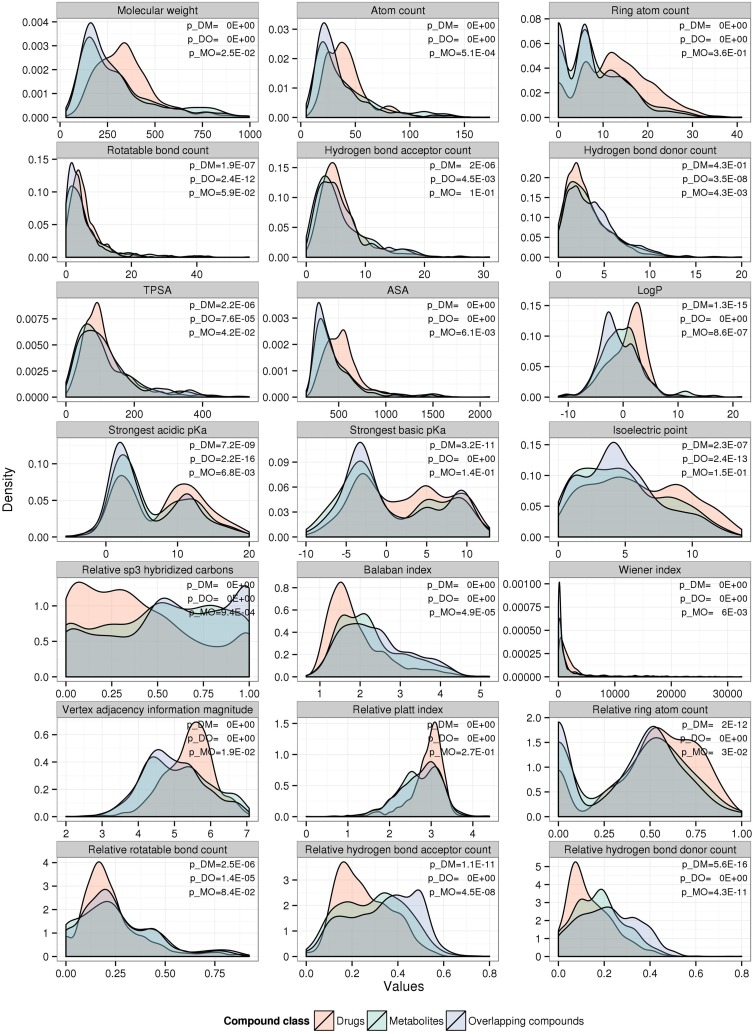
**Compound-class specific density distributions of various physicochemical properties**. The density plots were generated separately for drugs (red), metabolites (green), and overlapping compounds (blue). Statistical significance (*p*-value) was computed for drugs vs. metabolites (p_DM), drugs vs. overlapping compounds (p_DO), and metabolites vs. overlapping compounds (p_MO) by Kolmogorov–Smirnov test.

In order to characterize metabolites, drugs, and overlapping compounds with regard to specific physicochemical properties governing their protein binding behavior, we computed a range of relevant properties typically used in the field of cheminformatics (Supplementary Table 1 contains a list along with definitions) for all compounds in the respective sets and tested them for significant frequency distribution differences using the two-sample Kolmogorov-Smirnov test (Figure 1) (Lilliefors, 1967).

Across the set of physicochemical properties examined, drug compounds possess distinctive characteristics compared to both metabolites and overlapping compounds, whereas the set of compounds classified as both drugs and metabolites (overlapping compounds) are more similar to metabolites than to drugs (Figure 1). On average, the drug compounds used here are larger than metabolites with higher values for molecular weight (medians of 330.2Da vs. 238.7Da for drugs and metabolites, respectively, p_*Wilcox*_ = 1.2E-19), atom count (38 vs. 30, *p* = 6.7E-12), ring atom count (12 vs. 6, *p* = 2.0E-35), accessible surface area (ASA) (514.6Å^2^ vs. 394.4Å^2^, *p* = 3.7E-23), have fewer hydrogen bond donors (0.12 vs. 0.18, *p* = 1.7E-15), and acceptors (0.23 vs. 0.3, *p* = 5.2E-09) when normalized for size, and carry both weaker acidic and basic functional groups [higher strongest acidic (8.89 vs. 4.36, *p* = 9.7E-06) and basic (2.28 vs. −1.53, *p* = 4.4E-09) pK_a_] and can therefore be assumed less charged at physiological pH. Reduced polarity and charge of drugs is also mirrored by their increased hydrophobicity [higher logP (octanol partition coefficient)] relative to metabolites (1.43 vs. -0.3, *p* = 3.2E-13). A relatively large number of drugs appears to be positively charged at neutral pH (secondary peak of the isoelectric point distribution around pI = 9), while metabolites predominantly carry negative charges at neutral pH. The topological polar surface area (TPSA) appears similar for all compound classes (median of ~90 Å^2^). However, as drugs are, on average, bigger and have larger ASA, the reduced polarity of drugs relative to metabolites is evident again. Even though the mode of the relative rotatable bond count density distribution is similar for all three compound classes, drugs possess distinctly more ring atoms relative to their size (higher relative ring atom count: 0.56 vs. 0.46, *p* = 8.6E-18) and relatively fewer sp^3^-hybridized carbon atoms (0.33 vs. 0.53, *p* = 2.6E-16). Various graph-based measures have become popular in the field of cheminformatics to describe the topologies of compounds (see Supplementary Table 1 for brief descriptions). The Balaban index is smaller for drugs than for metabolites reflecting the increased ring atom count (1.69 vs. 2.12, *p* = 1.9E-29). Other graph indices are increased for drugs [Wiener index (1149 vs. 461, *p* = 8.9E-19), vertex adjacency information magnitude (5.46 vs. 5, *p* = 3.7E-19)]. However, as these indexes are positively correlated with atom count - in a non-linear fashion—the observed difference appears largely a consequence of size rather than topological differences. The normalized Platt index, the sum of the edge degrees of the graph representing the chemical structure of a compound divided by the number of atoms, reveals a similar mode of the distribution for all three compound classes, but a narrower distribution for drugs, while metabolites are more diverse in their topologies. Across all investigated properties, overlapping compounds show similar distributions as metabolites rather than drugs (Figure 1).

As drugs and metabolites display distinct physicochemical property profiles (Figure 1), it seems possible to classify them using those properties as predictor variables. Applying a classification and regression tree algorithm (*rpart* R-package), prediction of compound class was possible, albeit with limited purity (28.5% error rate for models with (without) size-dependent properties, Supplementary Figure 1). As already implied by the observed property profiles ASA, logP, and relative sp^3^-hybridized carbons proved as most informative predictors.

### Characterization of compound binding promiscuity

Next, we explored, which physicochemical properties impart compound binding promiscuity vs. selectivity and whether these properties may be different for metabolites and drugs.

**Figure 2:**
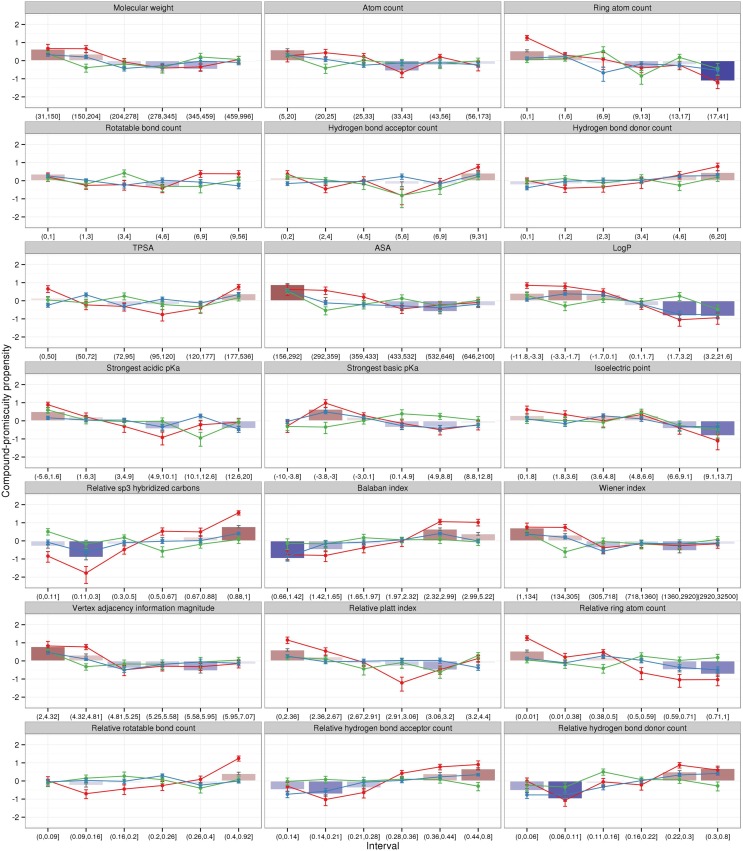
**Logarithmic promiscuity propensity ratios of all compounds (bars) and individual compound classes (lines) for diverse physicochemical properties**. Positive propensity values (red color gradient) denote that a given property interval is characteristic for promiscuous compounds. Negative values (blue color gradient) show that a property interval is biased in favor of selective compounds, which have only one or two target pockets. Differently colored lines and associated error bars correspond to drugs (red), metabolites (green), and overlapping compounds (blue). Error bars denote the estimated standard error of the mean values.

For the set of different physicochemical properties characterized above, we tested whether compounds associated with a particular value range are more likely specific (fewer than three binding pockets) or promiscuous (three or more binding pockets) expressed as propensity values. Positive values denote that a particular property and interval range is likely associated with promiscuous compounds and negative values are preferably found for selective compounds (see Materials and Methods). All 2886 compounds were tested as a combined set as well as for drugs, metabolites, and overlapping compounds separately (Figure 2).

For the combined compound set, all properties generally follow a monotonic trend with regard to being associated with either selective or promiscuous binding behavior (bars in Figure 2). Small values are associated with promiscuity for properties molecular weight (<150 Da), atom count (<20), ring atom count (<6), accessible surface area (<292 A^2^), logP (<0.1), strongest acidic (<1.6), or basic (<-3) pK_a_, vertex adjacency information magnitude (<4.81), Wiener index (<305), and relative ring atom count (<0.01). Conversely, large values of the same property are associated with selective binding behavior. The opposite trend (small values indicative of selective and large values of promiscuous behavior) is apparent for the properties (with threshold values indicating promiscuous binding) hydrogen bond donor count (>4), relative sp^3^ hybridized carbons (>0.67), Balaban index (>2.32), relative rotatable bond count (>0.4), relative hydrogen bond acceptor (>0.36)/donor (>0.22) count. In addition, high isoelectric points (>6.6) appears to promote selectivity.

When inspected separately for the three compound classes (lines in Figure 2), drugs stand out as exhibiting the most pronounced propensity profiles across all properties with largest absolute propensity values compared to both metabolites and overlapping compounds with more shallower profiles. Unlike the monotonic profiles observed for the whole compound set, drugs display minimum/maximum propensity curves for several properties. As drugs can be assumed to have been selected specifically against high promiscuity, the minima for molecular weight (278–459 Da), TPSA (topological polar surface area around, 95–120 A^2^), strongest acidic pK_a_ (4.9–10.1), relative sp^3^ hybridized carbons (0.11–0.3), relative Platt index (2.91–3.06), relative rotatable bonds (0.09–0.16), relative hydrogen bond acceptor (0.14–0.21)/donor (0.06–0.11) count may correspond to optimal physicochemical properties imparting selectivity.

In summary, promiscuous compounds with many binding divers events observed in the PDB tend to be rather small, hydrophilic, and of low complexity allowing a good fit to more diverse and small binding pockets. Also a flexible backbone (e.g., high relative rotatable bond count and high sp^3^-hybridization level) enhances the ability of compounds to bind to different target pockets. In addition, the increased number of hydrogen bond acceptors and donors in those compounds is advantageous for formation of interactions with target proteins. Drug compounds exhibit more pronounced property propensities with regard to their promiscuity revealing also “sweet spots” associated with selective binding behavior. By contrast, metabolites and overlapping compounds exhibit shallow profiles with almost no apparent correlation with promiscuity.

### LogP and compound binding promiscuity

For metabolites, no dependency of binding promiscuity on compound hydrophobicity as measured by logP was detected, whereas for drugs, our analysis suggests that increasing hydrophobicity is negatively correlated with promiscuity (Figure 2, LogP), which is contrary to literature reports that describe hydrophobic drugs as less selective regarding their binding to proteins (Peters, 2013). To further scrutinize our result, we analyzed the relation between hydrophobicity (logP) and promiscuity (pocket count) for the different compound classes using all 2886 compounds and only those that are promiscuous (three or more binding pockets).

**Figure 3:**
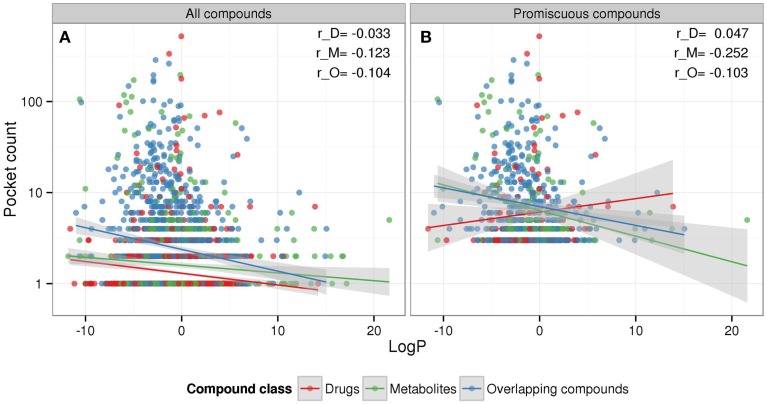
**Compound-type specific relationships between hydrophobicity (logP) and promiscuity (pocket count)**. The scatter plots show the three compound classes drugs (red), metabolites (green), and overlapping compounds (blue) including their linear regression curves and 95% confidence region (gray) for **(A)** both selective and promiscuous compounds together and **(B)** promiscuous compounds only with at least three non-redundant target pockets. Corresponding Pearson correlation coefficients for drugs (r_D), metabolites (r_M), and overlapping compounds (r_O) are also displayed.

Considering all compounds (selective and promiscuous compounds), hydrophobicity and promiscuity are negatively correlated for all three compound classes, albeit at very low correlation coefficient levels (Figure 3). By contrast, using promiscuous compounds only, drugs show a weak positive correlation, which is in agreement with literature, whereas metabolites maintain a negative correlation, which is significantly different (*p* = 0.0026) compared to drugs (Supplementary Figure 2). Thus, the reported dependency of binding behavior on logP may be set-dependent (see Discussion). Again, as seen above (Figure 2), drugs and metabolites display distinctive relationships of physicochemical properties and binding behavior.

### Protein target-centric investigation of binding events

So far, we focused on compound properties relevant for their interaction with proteins. Next, we shall examine the characteristics of their cognate proteins, and specifically, of the binding pockets/sites involved in the physical compound-protein binding event. Again, we wished to examine whether metabolites and drugs are associated with similar or different binding pocket properties and whether binding sites of promiscuous compounds are different from those bound by specific compounds.

We determined the amino acid composition of binding pockets relative to non-binding site regions of proteins and computed composition propensity values (see Materials and Methods) of binding pockets dependent on bound compound class and compound promiscuity using 12,422 protein pockets interacting with the 2886 compounds (see Table 1B). Positive propensity values represent a bias of specific amino acid residue types to occur more frequently in binding pockets, while amino acid residues with negative composition propensity are less frequent in binding pockets than in other parts of proteins.

**Figure 4:**
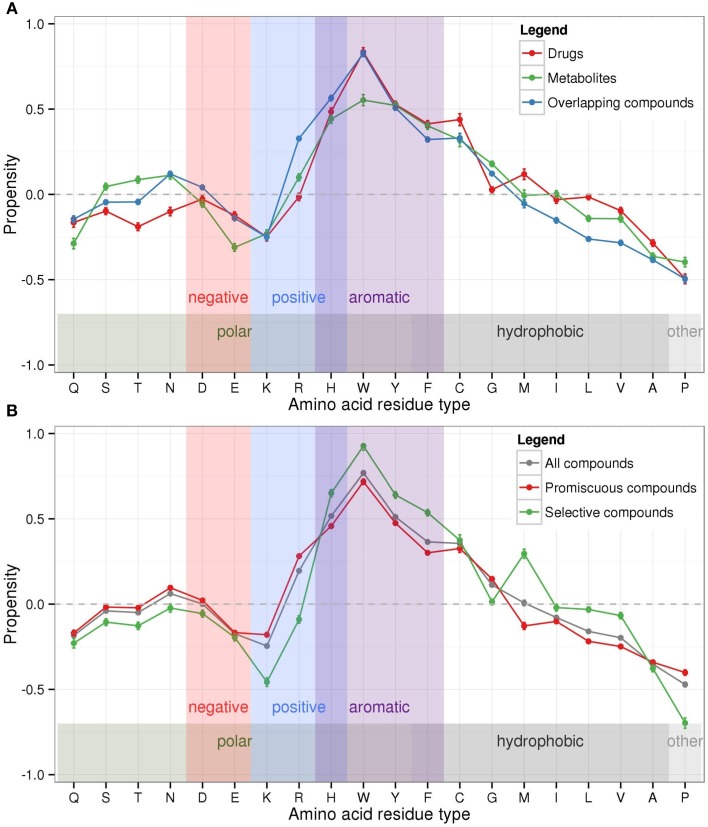
**Logarithmic propensities of amino acid binding pocket composition**. Propensities were calculated for the amino acid composition of binding pockets in relation to other protein regions with respect to **(A)** the three bound compound classes drugs (red), metabolites (green), and overlapping compounds (blue), and **(B)** binding pockets associated with all bound compounds (gray), promiscuous compounds (red), and selective compounds (green), respectively. The background shading refers to the physicochemical properties of amino acids according to Taylor (1986). Error bars denote the estimated standard error of the mean values. (Connecting lines between propensity values serve improved traceability only).

Aromatic amino acids (histidine-H, phenylalanine-F, tryptophan-W, and tyrosine-Y) tend to occur more frequently in binding pockets than in other protein regions, which was also shown by Binkowski et al. (2003) and explained—at least in part—by the observed high catalytic propensity of histidine and tryptophan (Bartlett et al., 2002) (Figure 4A). Of the charged amino acid residue types, arginine (R) appears preferred, glutamate (E), and lysine (K) depleted, while aspartate (D) seems indifferent with regard to their propensity to occur in binding sites. Cysteine (C) occur more frequently in binding pockets, while other small hydrophobic amino acids (alanine-A, valine-V, leucine-L) occur less often than expected. Proline (P) was found to be least preferred binding pockets. Other polar or hydrophobic residues (serine-S, threonine-T, asparagine-N, glycine-G, methionine-M, isoleucine-I) show inconsistent preferences (across all compound classes) for binding pocket locations.

Overall, the three different compound classes display similar compositional propensity profiles (Figure 4A). Noteworthy differences between drugs and metabolites are evident for polar amino acids with metabolite-binding sites showing increased frequencies (serine-S, threonine-T, asparagine-N), while drug-sites show depleted levels. Tryptophan (W) is found relatively more often in drug-sites than in metabolite-binding sites, with the latter showing a bias against negatively charged glutamate (E) compared to drug-sites. Surprisingly, overlapping compounds appear to display a preference for binding sites with depleted frequencies of branched hydrophobic amino acid types (isoleucine-I, leucine-L, and valine-V).

The amino acid composition propensities calculated for protein sites bound by either selective or promiscuous compounds follow similar general trends as described above (Figure 4B). Nonetheless, small but significant differences are apparent between the two compound categories. Protein binding sites interacting with selective compounds are associated with more pronounced amino acid propensities (larger values) than sites binding promiscuous compounds. Selective compounds tend to bind to pockets with increased frequencies of aromatic residues and methionine (M) in their binding pockets, but decreased occurrences of polar and positively charged amino acid residue types and depleted proline (P). By contrast, promiscuous compounds display a preference for sites with decreased (branched) hydrophobic residues (methionine-M, isoleucine-I, leucine-L, valine-V). The propensity profile of sites binding selective compounds is more similar to that of drugs (correlation coefficient between the two profiles *r* = 0.98) rather than metabolites (*r* = 0.91) and overlapping compounds (*r* = 0.89) (Figure 4A). This similarity of profiles is consistent with the notion that drugs are rather selective, which fits the requirements of a targeted pharmaceutical intervention (Peters, 2013). Please note that the displayed error bars in Figure 4 representing the estimated errors of mean values are very small because of high counts entering the calculation.

### Functional and compositional diversity of target proteins and binding sites

After examining general amino acid propensities in binding pockets of proteins bound by the different compound classes and their promiscuity level, we studied the protein target diversity associated with promiscuous compounds based on the EC (Enzyme Commission) number classification scheme as well as on the amino acid composition of target pockets. While the EC-based diversity of targets captures its functional relevance from a metabolic viewpoint, the composition-associated diversity aims to establish whether promiscuity is caused by repeated use of the same binding site in otherwise different proteins (Haupt et al., 2013) or rather due to flexible binding modes to different target pockets. In the former scenario, pocket diversity would be low, while in the latter, it would be high for promiscuous compounds.

### Enzymatic biochemical target diversity, EC entropy

For every compound from all three compound classes, we calculated its EC entropy, *H*, based on the six top-level EC numbers that classify enzymes by the reactions they catalyze, e.g., enzymes with “EC 1” represent oxidoreductases, with “EC 2” transferases, “EC 3” hydrolases, “EC 4” lyases, “EC 5” isomerases and “EC 6” ligases, where the label “None” was introduced for proteins without EC number assignment (see Materials and Methods).

**Figure 5:**
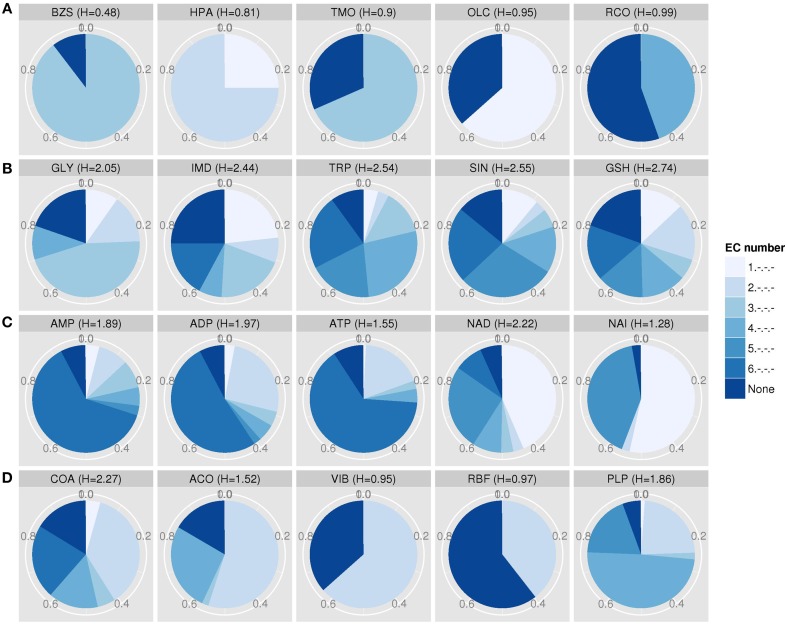
**EC entropies of metabolites with at least five target proteins. (A)** The top five metabolites with the lowest EC entropy: benzylsuccinate (PDB ID: BZS), hypoxanthine (HPA), trimethylamine N-oxide (TMO), oleoylglycerol (OLC), and resorcinol (RCO). **(B)** The bottom five metabolites with highest entropy: Glycine (GLY), imidazole (IMD), tryptophan (TRP), succinate (SIN), and glutathione (GSH). **(C)** The general energy currency metabolites adenosine mono-, di- and triphosphate (AMP, ADP, ATP) and redox equivalents NAD (NAD) and NADH (NAI). **(D)** The cofactors and vitamins coenzyme A (COA), acetyl- coenzyme A (ACO), thiamine (VIB, vitamin B1), riboflavin (RBF, vitamin B2), and pyridoxal-5′-phosphate (PLP, vitamin B6 phosphate).

Compounds with low EC entropy show a preference for specific enzyme biochemical classes, while those with high EC entropy bind to proteins engaging in a broader range of enzymatic reaction types. In the following, we shall discuss a few selected biologically relevant metabolites and those with extreme entropy values. Their EC diversity is also displayed graphically (Figure 5). Benzylsuccinate (PDB ID: BZS) was detected with very low entropy (*H* = 0.48) and binds mainly to enzymes with the EC class “3,” i.e., hydrolases. In fact, BZS is described as an intermediate in benzoate degradation, which can be converted to benzylsuccinyl-CoA via the enzyme benzylsuccinate CoA-transferase and is classified as a transferase (EC 2.8.3.15) (Leutwein and Heider, 2001). Hypoxanthine (HPA) is very specific as well and prefers oxidoreductases (EC 1) and transferases (EC 2) as targets. The metabolite trimethylamine N-oxide (TMO), an oxidation product of trimethylamine catalyzed by the enzyme dimethylaniline monooxygenase (Treacy et al., 1998) (EC 1), binds preferably to hydrolases (EC 3). Further, oleoylglycerol (OLC) and resorcinol (RCO) have low EC entropy and target oxidoreductases (EC 1) and lyases (EC 4), respectively. Being associated with a high percentage (>30%) of target proteins without EC classification, the metabolites TMO, OLC, and RCO bind also to proteins without catalytic function like membrane proteins (Efremov and Sazanov, 2012), hormones (Tang et al., 1999), or to enzymes, which are not yet classified. By contrast, the amino acids glycine (GLY) and tryptophan (TRP) interact nearly equally with every EC class enzyme. This applies also to succinate (SIN), a common organic acid, imidazole (IMD), and glutathione (GSH), an important antioxidant and redox-state regulator. The so-called energy currency metabolites adenosine mono-, di- and triphosphate (AMP, ADP, ATP) have a medium entropy and bind to all enzymes classes, but also show a preference for ligases, which catalyze the formation molecular bonds upon hydrolyzing ATP. NAD (NAD, nicotinamide adenine dinucleotide) and NADH (NAI, reduced form of NAD) preferably bind to enzymes catalyzing oxidations or reductions, which in turn are often accompanied by such redox equivalents, but have also a preference for isomerases (EC 4). NAD has a broader EC range than NAI. The cofactors coenzyme A (COA) and acetyl- coenzyme A (ACO) bind mainly to transferases (EC 2), whereby COA frequently also binds to all other enzyme classes and ACO to lyases (EC 4). Thiamine (vitamin B1, VIB) and riboflavin (vitamin B2, RBF) are involved in reactions catalyzed by transferases (EC 2). Lastly, pyridoxal 5′-phosphate (PLP), also known as B6 vitamin phosphate, interacts with lyases (EC 4), transferases (EC 2), and oxidoreductases (EC 1) (listed in decreasing percentage order of EC classes).

### Protein binding pocket variability

We assessed the diversity of binding pockets associated with every compound. As a metric of pocket diversity, we used a measure of amino acid compositional variation, the pocket variability, *PV* (see Materials and Methods).

**Figure 6:**
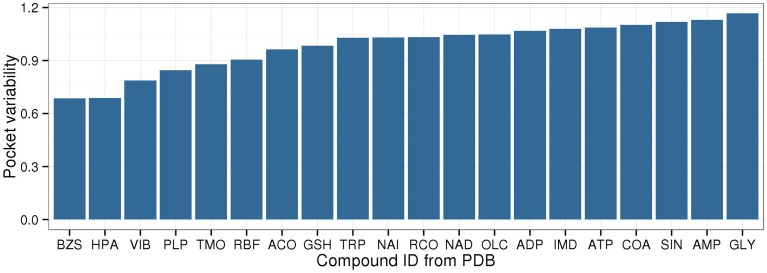
**Binding pocket variability for metabolites with at least five target pockets**. The same set of metabolites is displayed as in Figure 5, showing the top/bottom five metabolites with lowest/highest EC entropy, the energy currencies, redox equivalents, cofactors, and vitamins.

Among the 20 selected compounds presented in Figure 5, the largest PVs were determined for succinate (SIN), AMP, and glycine (GLY), while the smallest PVs were found for benzylsuccinate (BZS), hypoxanthine (HPA), and thiamine (VIB) (Figure 6).

**Figure 7:**
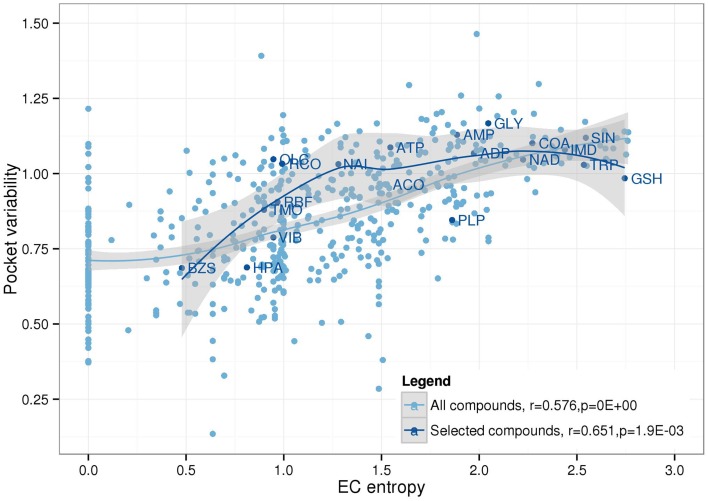
**Relationship between EC entropy and pocket variability**. Linear Pearson correlation coefficients and associated *p*-values were calculated for all compounds (lightblue) and the 20 selected compounds (darkblue) as displayed in Figure 5. Loess function was used to smooth the distribution (lines) including a 95% confidence region (gray).

As can be expected, there is an overall positive correlation between PV and EC entropy (Figure 7). Compounds that tolerate different binding pockets as judged by their amino acid residue compositional diversity can bind to more proteins allowing a broader EC spectrum. Thus, from high PV, high EC entropy follows naturally as observed for the nucleotides AMP, ADP, ATP, or the amino acid glycine. By contrast, low PV should generally be associated with low EC entropy as indeed detected for benzylsuccinate (BZS) and hypoxanthine (HPA). However, it is conceivable that some compounds have stringent binding pocket requirements (low PV), but the preferred binding pocket is found on many different proteins involved in different enzymatic processes entailing high EC entropy. For example, glutathione (GSH) and pyridoxal-5′-phosphate (PLP) have relatively low PV, but high EC entropy and fall into this category. By contrast, high PV and associated low EC entropy should be associated with compounds that have a specific biochemical role, but tolerate different binding sites. Decanoic acid (DKA) and 1-Hexadecanoyl-2- (9Z-octadecenoyl)-sn-glycero-3-phospho-sn-glycerol (PGV), both lipid associated metabolites exhibit this behavior.

Table 2 shows all 4 combinations PV (high/low), EC entropy (high/low) and representative compounds falling into the respective categories taking from the whole compound sets.

**Table 2:** **Compounds with extreme pocket variability (PV) and enzymatic target diversity (EC entropy) and combinations thereof**.

	**EC high (> = 2)**	**EC low (<1)**
PV high (> = 1.2)	Guanosine-5′-monophosphate (5GP), bis (adenosine)-5′-tetraphosphate (B4P), Guanosine-5′-triphosphate (GTP), Palmitic acid (PLM)	Decanoic acid (DKA), 1-Hexadecanoyl-2- (9Z-octadecenoyl)-sn-glycero-3-phospho-sn-glycerol (PGV)
PV low (<0.8)	Fructose-1,6-biphoshate (FBP), Oxamic acid (OXM)	172 compounds

*Thresholds were chosen arbitrarily to retrieve a small number of exemplary compounds derived from the whole compound set*.

On average, among the sets of compounds used in this study, drugs have lower EC entropy and pocket variability than metabolites or overlapping compounds (Table 3), albeit significance could not be generally established (*t*-test *p*-values for the comparison of drugs vs. metabolites/overlapping compounds, EC entropy: 0.09/2.16E-03, PV: 0.15/3.03E-04). This indicates again the higher specificity of drug-target interactions, not only from the compound side, but also from the protein target side.

**Table 3:** **Compound-type specific target protein diversity**.

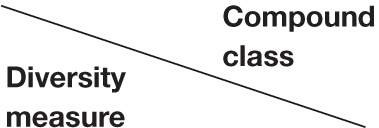	**Drugs**	**Metabolites**	**Overlapping compounds**
Enzymatic target diversity, EC entropy	0.900 (0.746)	1.080 (0.696)	1.183 (0.681)
Pocket variability, PV	0.776 (0.220)	0.816 (0.198)	0.860 (0.187)

*EC entropies and pocket variabilities were calculated for each compound separately and averaged across all compounds of identical class (drug, metabolite, overlapping compound). Listed are the respective mean values with associated standard deviations in parentheses*.

### Prediction of compound promiscuity using physicochemical properties

Predicting compound selectivity/promiscuity is a central goal in cheminformatics. We applied Partial Least Square regression (PLSR) and Support Vector Machines (SVMs) to predict from physicochemical properties both the number of different binding pockets and the tolerance to bind to different binding pockets as measured by the pocket variability. Applying PLSR allows for the prediction of a continuous outcome variable and efficient handling of correlated predictor variables, while SVM was used for the binary promiscuous/selective call and allows applying non-linear functional relationships between predictor and target variables. The models were generated for all compounds jointly and the three compound classes drugs, metabolites, and overlapping compounds separately.

**Figure 8:**
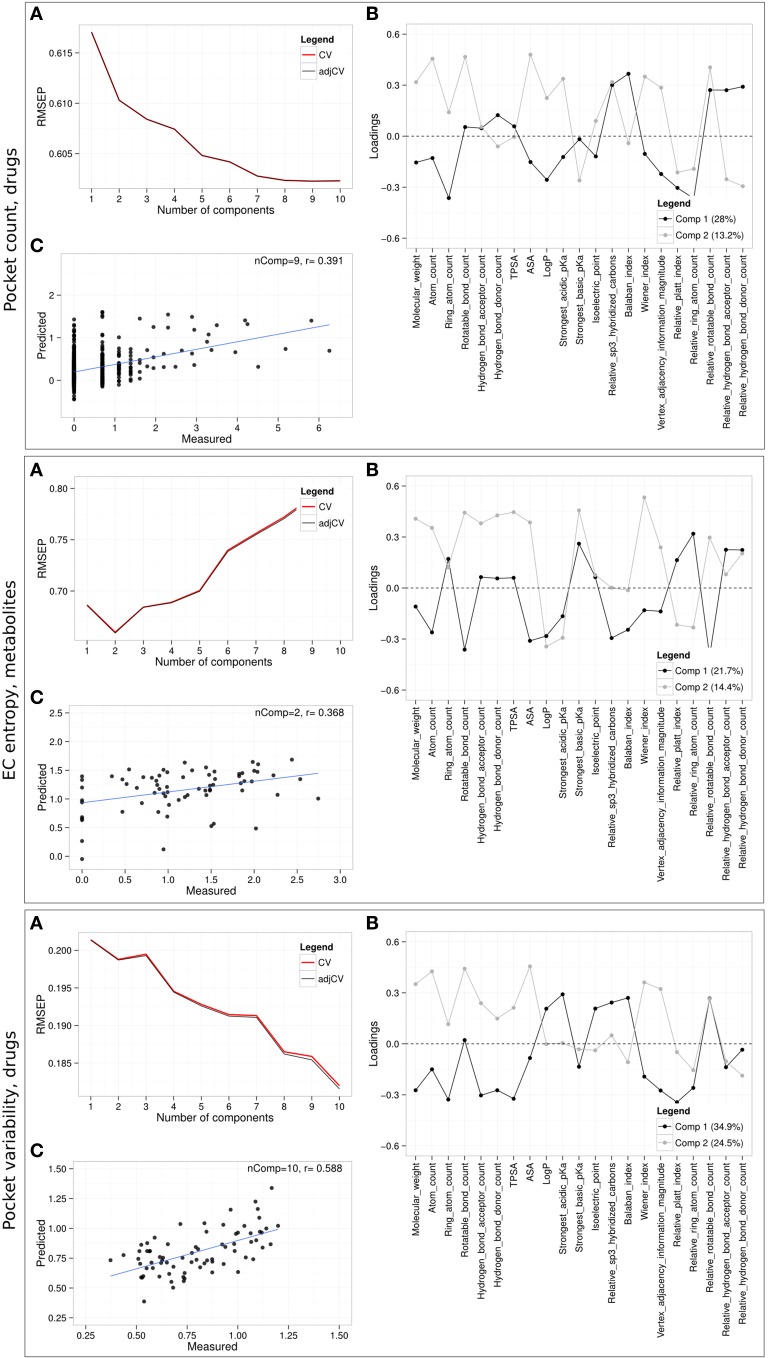
**Partial least squares regression (PLSR) using physicochemical properties**. PLSR prediction models were built for drug promiscuity (logarithmic pocket count), drug pocket variability and EC entropy of metabolites. **(A)** Cross-validated (CV) RMSEP (root mean square error of prediction and adjusted CV) curves as function of the number of components in the model, **(B)** loading plot of the physicochemical properties for the first two components, and **(C)** measured against predicted values including the number of components used in the final prediction model (nComp) and correlation coefficient, r, in a leave-one-out cross-validation setting. PLS models for the respective additional compound classes resulting in inferior performance relative to the one shown here are presented in Supplementary Figures 3, 4.

Regarding the predictability of promiscuity captured by target pocket count, best results were achieved for drugs (Figure 8, “Pocket count, drugs”) with nine principal components (nComp = 9) and a Pearson correlation coefficient of 0.391 between measured and predicted pocket counts in a leave-one-out cross-validation setting. The associated loadings that indicate how much a physicochemical property contributes to the prediction of pocket count associated with the first component show high covariances for Balaban index, relative hydrogen bond acceptor and donor count, sp^3^-hybridization level and relative rotatable bond count. The latter two properties capture compound flexibility found to be positively correlated with promiscuity. Large negative loadings on the first component comprise the properties ring atom count, logP, relative Platt index and relative ring atom count. Although the predictive models for metabolites, overlapping compounds, and all compounds taken together resulted in only modest correlations of measured to predicted pocket counts (*r* = 0.2, 0.303, 0.364, respectively), the tendencies of the first component loadings were similar as for drugs, whereas those of the second component differ for each compound class (Supplementary Figure 3).

Similar prediction results were obtained for EC entropy as the chosen target variable with comparable correlations of measured to predicted pocket variabilities for all compounds (*r* = 0.342), drugs (*r* = 0.324), metabolites (*r* = 0.368), and overlapping compounds (*r* = 0.327) (Figure 8, “EC entropy, metabolites” and Supplementary Figure 4).

While the resulting PLS model for pocket variability, *PV*, yielded poor correlations of measured and predicted values for all compounds, metabolites, and overlapping compounds (r_all_ = 0.246, r_M_ = −0.04, r_*O*_ = 0.095), the model for drugs returned good results with a high correlation (*r* = 0.588) between measured and predicted values (Figure 8, “Pocket variability, drugs”). Large positive loadings of the first component indicate high covariances with *PV* of logP, strongest acidic pK_a_, isoelectric point, relative sp^3^-hybridization, Balaban index, and relative rotatable bond count. Negative loadings were associated with size- and complexity dependent descriptors (molecular weight, ring atom count, hydrogen acceptor/donor count, TPSA, Wiener index, Vertex adjacency information magnitude) as well as other descriptors such as relative Platt index and relative ring atom count.

We also applied SVMs for the binary classification of compounds into promiscuous vs. selective binding behavior. Unlike the linear PLS approach, SVMs allow for non-linear relationships as may appear promising given the non-linear relationships of selected properties with promiscuity, especially for drugs (Figure 8). However, performance in cross-validation was similar across various applied linear and non-linear kernel functions (Supplementary Table 3). The lowest cross-validation error for drugs was determined at 26.1%, while it was 44.3% for metabolites. For comparison, random predictions would result in 50% error.

Taken together and in line with previous reports (Sturm et al., 2012), the set of physicochemical properties used here proved informative for the prediction of target diversity and compound promiscuity with properties capturing flexibility (relative rotatable bond count and sp^3^-hybridization level) and hydrogen-bond formation descriptors (relative hydrogen bond acceptor and donor count) being most predictive, albeit prediction accuracies reached modest accuracy levels only. Prediction models were consistently better for drugs than for metabolites, reflected already by the more pronounced correlation of the various physicochemical properties and promiscuity (Figure 2).

### Metabolite pathway, process, and organismal systems enrichment analysis

To investigate whether selective or promiscuous metabolites serve specific biological functions, we performed an enrichment analysis using pathway maps obtained from the KEGG pathway database (http://www.genome.jp/kegg/pathway.html). We used collective and detailed pathway ontologies for the categories “Metabolism,” “Environmental Information Processing,” and “Organismal Systems,” to which the metabolites were assigned using chemical structure fingerprints (see Materials and Methods), and calculated the significance of enrichment and depletion for the set of promiscuous and selective metabolites by applying the Fisher's exact test (Table 4).

**Table 4:** **Metabolite pathway, process, organismal system ontology enrichment with respect to compound promiscuity**.

	**Promiscuous metabolites**		**Selective metabolites**	
	**P_FDR_-value**	**Pathway name**	**P_FDR_-value**	**Pathway name**
**METABOLISM**				
Collective	4.96E-02	Energy metabolism	6.72E-02	Carbohydrate metabolism
	4.96E-02	Nucleotide metabolism	9.06E-02	Metabolism of terpenoids and polyketides
	7.73E-02	Amino acid metabolism		
Detailed			6.69E-02	Polyketide sugar unit biosynthesis
	**P_FDR_-value**	**Process**	**P_FDR_-value**	**Process**
**ENVIRONMENTAL INFORMATION PROCESSING**				
Collective	6.79E-03	Signal transduction	1.63E-03	Not assigned
Detailed	3.14E-02	AMPK signaling pathway	1.94E-05	Not assigned
	4.52E-02	HIF-1 signaling pathway		
	**P_FDR_-value**	**System**	**P_FDR_-value**	**System**
**ORGANISMAL SYSTEMS**				
Collective	4.41E-05	Digestive system	1.67E-11	Not assigned
	5.42E-04	Nervous system		
				
Detailed	2.68E-02	Vitamin digestion and absorption	3.05E-13	Not assigned
	7.64E-02	Synaptic vesicle cycle		

*Enrichment analysis was performed for “Metabolism,” “Environmental Information Processing,” and “Organismal Systems” categories using both collective and detailed ontology terms obtained from the KEGG pathway database. Displayed are the enriched pathways for promiscuous and selective metabolites with Benjamini-Hochberg procedure corrected p-values (<0.1). Note that the category “Not assigned” was introduced for all metabolites lacking a specific annotation in the respective category*.

Regarding metabolism, promiscuous metabolites were found enriched in energy, nucleotide, and amino acid metabolism pathways. Among the 14 promiscuous metabolites associated with energy pathways were energy currency compounds and redox equivalents ADP, ATP, NADH, NAD+ as well as the central metabolites pyruvate, succinate, and the amino acid glycine. Partly overlapping with energy metabolism, promiscuous compounds were also found associated with nucleotide metabolism. The AMP, ADP, ATP, dAMP, dGMP, glycine were among those metabolites. By contrast, selective compounds were preferentially found in carbohydrate metabolic processes, which are predominantly sugar derivatives, as well as metabolic processes involving terpenoids and polyketides including sugar derivatives/phosphates as dTDP-4-amino-4,6-dideoxyglucose (0FX) or dTDP-4-oxo-2,6-dideoxy-D-glucose (DWN), but also abscisic acid (A8S), which is a central plant hormone involved in many plant development processes. Correspondingly, the term “Polyketide sugar unit biosynthesis” was found enriched among in detailed term list for selective metabolites.

In the environmental KEGG category, promiscuous metabolites were detected significantly enriched in signal transduction pathways comprising both general energy currency metabolites and more specific compounds such as serotonin (SRO)—a common neurotransmitter, zeatin (ZEA)—a cytokinin acting as a plant growth hormone, and phytate (IHP)—an important phosphorus storage in plants. Supplementary Figure 5 shows the chemical structure of those three compounds. Phytate has been reported to also have roles in neurotransmission (Vallejo et al., 1987), in protein activation or inhibition (Efanov et al., 1997; Larsson et al., 1997), in the process of DNA reparation (Hanakahi et al., 2000) or in mRNA export from the nucleus to the cytosol (York, 1999), and other processes (Shears, 2001). The AMPK signaling pathway, in particular, is enriched with promiscuous compounds.

Regarding organismal systems, promiscuous metabolites were found enriched in the digestive (e.g., the metabolites choline, serotonin, glutathione, pantothenate, vitamins A, B1, and others) and nervous systems (e.g., ATP, choline, succinate, acetyl-CoA (ACO), histamine and others). More specifically, promiscuous metabolites were detected associated with vitamin digestion and absorption pathways.

As a set, selective metabolites were not found specifically enriched in any environmental or organismal system. This result seems expected as specific metabolites are by definition less likely to accumulate in specific processes as they are only bound to very few target proteins/enzymes.

In summary, promiscuous metabolites found associated with specific pathway enrichments in the “Metabolism,” “Environmental Information Processing,” and “Organismal systems” categories are mainly energy currency compounds, redox equivalents, cofactors or vitamins and other amino acids. Thus, although promiscuous, they can be found preferentially in specific metabolic and signaling processes. By contrast, despite their reduced promiscuity, as a set, selective metabolites do not accumulate in specific pathways, but are found across many different metabolic processes. Noticeable exception is the carbohydrate metabolism with mainly selective sugar derivatives. A detailed overview of all metabolite sets and their pathway associations is provided Supplementary Table 2.

## Discussion

We performed a systematic comparative analysis of metabolite and drug compound sets regarding their physicochemical properties and associated protein binding promiscuity. It may be questioned whether making a distinction between metabolites and drugs with regard to their binding behavior is reasonable. After all, both are sets of small chemicals whose interactions with other molecules ought to be governed by the same physicochemical principles. However, drugs constitute a special class of compounds that were man-selected for a particular purpose. Therefore, the relationships of physicochemical properties and binding behavior reported for drugs may neither be representative for all compounds in general nor metabolites in particular. Furthermore, metabolites have their own specific functional implications, i.e., to be involved in enzymatic reactions. Thus, phenomena related to enzymatic diversity are relevant for metabolites, but not necessarily for drugs. Indeed, we found significant differences not only with regard to property profiles (Figure 1), but also concerning the association of properties and binding behavior (Figure 2). Drugs exhibit pronounced dependencies, whereas metabolites show much weaker correlations of properties and binding promiscuity. While reasonably successful for drugs, predicting promiscuous metabolite binding behavior proved less reliable (Figure 8, Supplementary Figures 3, 4). Again, because the governing physicochemical principles can be assumed identical, drugs should be regarded as a special subset in chemical space. As they have been selected for their very property of binding selectively to reduce adverse side effects, departures from this behavior resulting in promiscuous binding can be attributed to distinct physicochemical properties. By contrast, metabolites function both as selective and promiscuous compounds. As our results suggest, both binding characteristics can be accomplished by compounds of diverse physicochemical characters. Very likely, the evolutionary selection pressure acting on metabolites mediated by the evolutionary forces that shaped the organismic genomes and the set of encoded enzymes operated under constraints other than those proving ideal for drugs and their protein interaction range. Therefore, our results also imply that protein binding prediction results obtained for a particular compound class cannot be transferred directly to others. Evidently, our results are valid of the set of physicochemical properties selected here, albeit a broad range of different parameters was included in this study. Conceivable alternative properties may result in different conclusions.

Despite the marked differences of binding characteristics between the metabolite and drug compound sets, including both compound classes in a joint analysis may still prove useful toward achieving the goal of building prediction models of binding specificity. Rather than whole-compound based approaches, the concept of breaking down structures into sets of distinct pharmacophores and functional chemical groups and investigating their protein binding preferences may prove useful (Meslamani et al., 2012). It can be expected that the inclusion of as many compounds as possible regardless of the compound-class will help establishing statistical robustness.

We based our analysis on the comprehensive structural information on protein-compound interactions present in the PDB and the subsequent classification of bound compounds into drugs and metabolites with the aid of the public data resources DrugBank, ChEBI, HMDB, and MetaCyc. While successful in generating a dataset of sufficient size for the investigation of similarities and differences of compound classes and their promiscuity, it must be cautioned, however, that the PDB is not free from selection bias, in particular with regard to selection of protein type and covered enzyme classes (Mestres, 2005). However, as we implemented very strict requirements on tolerated sequence and structural similarities of proteins and binding pockets respectively, improper bias from redundancy seems safely excluded. Alternative approaches toward assessing binding promiscuity are conceivable. For example, a metabolite's promiscuity could also be gauged as the number of diverse chemical reactions it is involved in. However, we wished to base our study specifically on structurally determined binding events as this approach allowed to better cope with binding mode redundancy, which is unclear from chemical reaction annotations alone.

Another cautionary remark is warranted regarding the binding affinities of compounds contained in the PDB to their respective target protein. Depending on compound solubilities and concentrations as well as the experimental conditions applied during crystallization, binding affinities (K_d_) can be relatively high (up to 10^2^μmol/l). However, for a set of 367 compound-target interactions used in this study for which binding affinities to the actual proteins have been reported in BindingDB (Liu et al., 2007), the median binding affinity is K_d_ = 0.21 μmol/l and third quartile (75% of all compounds with known K_d_) with K_d_ <8.2 μmol/l. Furthermore, by requiring close physical contacts between at least three separate amino acid residues with any given compound to be included in this study and therefore, in effect, filtering for large interaction surfaces, loose binding events will have been discarded. Thus, a large number of compound-protein interactions examined here can be assumed to correspond to tight binding events.

Despite the limitations of using the PDB, as ultimately, we wish to predict compound-protein binding events based on structural properties of both the ligand compound and the target protein, basing the survey presented here on structural information as captured in the PDB represents a necessary step toward achieving this goal.

For the profiling of drugs with regard to binding promiscuity, experimental binding assays such as the proprietary BioPrint database (http://www.cerep.fr) proved useful. Based on results obtained from BioPrint and also other studies, lipophilicity/hydophobicity (logP) was found positively correlated with increased promiscuity of drug compounds (Krejsa et al., 2003), while another study that also used PDB structures found no impact of hydrophobicity on promiscuity (Haupt et al., 2013). Across all compounds, we found logP to be weakly negatively correlated with promiscuity. However, when applied to promiscuous drug compounds only, i.e., grading the degree of promiscuity, but excluding selective compounds, a weak positive correlation was detected for drugs in line with previous reports (Figure 3). The observed differences may in part be explained by the use of the PDB as the data source, or may reflect that, indeed, the reported positive correlation of lipophilicity with binding promiscuity is not universally valid. Similarly, molecular weight is often but not always reported negatively correlated with promiscuity (Tarcsay and Keserű, 2013). We observed a negative correlation (Figure 3). Again, the same caveats on data sources apply. Furthermore, as seen before in the context of other properties, metabolites displayed a deviating association of logP and promiscuity maintaining an overall weak negative correlation, yet again underlining the differences between the compound classes examined.

For most physicochemical properties and value ranges studied here, an optimum curve exhibiting intervals of maximal/minimal propensity rather than monotonic relationship of property value and binding specificity was observed for drug compounds (Figure 2). Thus, the present study may help to guide the identification of lead compounds to fall into the sweet spot for desirable binding specificity.

On the technical side of our study, we based the mapping of compounds, which was necessary for the categorization of PDB compounds, on comparing fingerprints. Thus, our approach did not consider isomeric similarities such as stereoisomers or tautomers. Therefore, similar compounds such as diastereomers could be inadvertently mapped to each other, although they may have different physical and chemical properties. However, given the use of CDK-extended fingerprints (1024 bits), the frequency of false-positive matches can be expected to be small and, furthermore, most compound-related properties used here are relatively insensitive to isomeric and tautomeric differences.

In the present study, we made no distinction with regard to functional role of the actual binding site. In particular for metabolites, it is conceivable that the canonical binding of metabolites as substrates into their respective catalytic binding site on the enzymes acting on them may be subject to different constraints than auxiliary binding sites, e.g., allosteric sites.

The performed enrichment analysis on the association of promiscuous or selective metabolites with specific biological processes revealed that promiscuity may indeed possess a functional relevance. Promiscuous compounds, in particular, were found associated with specific processes (Table 4). Thus, as opposed to the desired selectivity of drug compounds, promiscuous binding may have proved evolutionarily advantageous. Here, the property of being universally usable as evident for energy currency metabolites such as ATP etc., may explain the observed tendency. However, as we also found signaling processes to be preferentially associated with promiscuous metabolites, of which the actual compounds proved to be known signaling molecules (Table 4, Supplementary Figure 5), suggests that broad protein targeting may have played a role in shaping molecular signaling processes deserving further investigation.

As the study of regulatory effects of metabolites mediated via specific binding events to signaling proteins is a central research question in functional metabolomics, we believe this comprehensive and systematic survey of metabolite-protein binding events may prove helpful for designing future studies on this subject.

## Author contributions

PK and DW conceived the study. PK performed all computational analyses. PK and DW analyzed and interpreted the data, and wrote the manuscript.

### Conflict of interest statement

The authors declare that the research was conducted in the absence of any commercial or financial relationships that could be construed as a potential conflict of interest.
