# Supplementary material for: Physicochemical characteristics of structurally determined metabolite-protein and drug-protein binding events with respect to binding specificity
Source: Front Mol Biosci. 2015 Sep 15;2:51. doi: 10.3389/fmolb.2015.00051 (PMC4569973; doi:10.3389/fmolb.2015.00051)
Supplement: Supplementary file 1 [file Presentation1.PDF]

Supplementary Information

"Physicochemical characteristics of structurally determined metabolite-protein and drug-protein binding events with respect to binding specificity."

Paula Korkuć and Dirk Walther

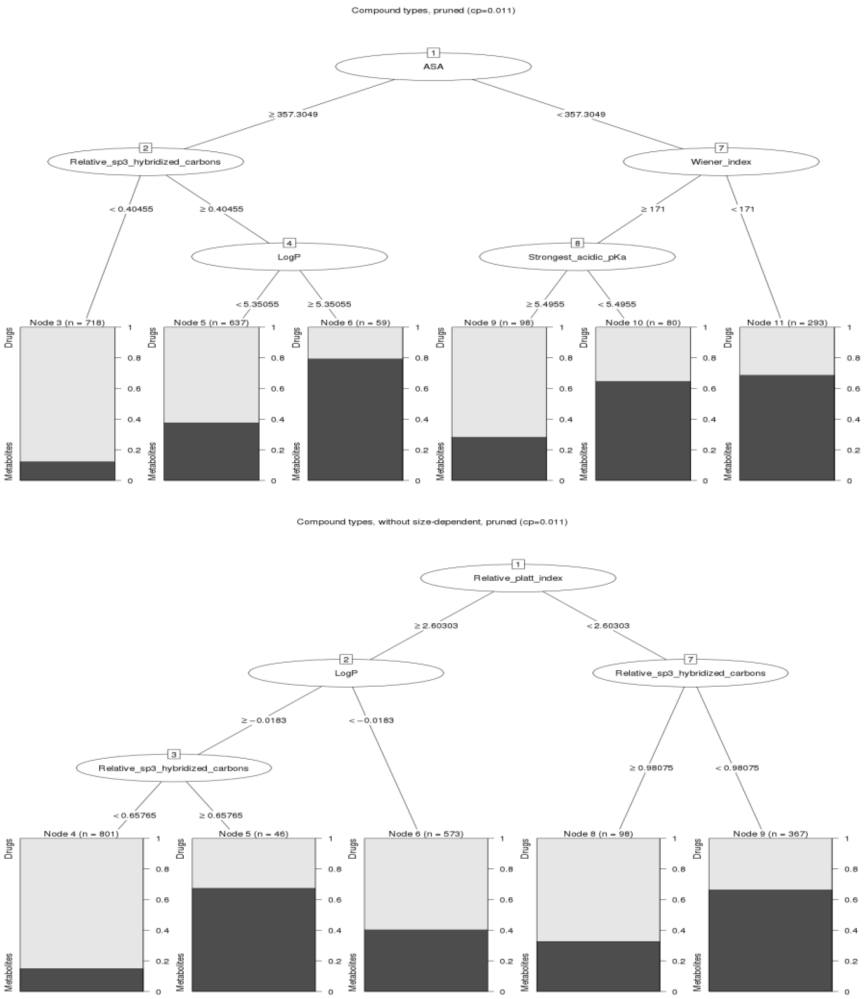

Supplementary Figure 1. Classification and regression tree for the prediction of compound class using the rpart R-package. A) All physicochemical properties examined in this study (cross-validated error rate=xerror*root node error=0.82*0.35=0.28), B) without size-dependent properties (0.83*0.35=0.29).

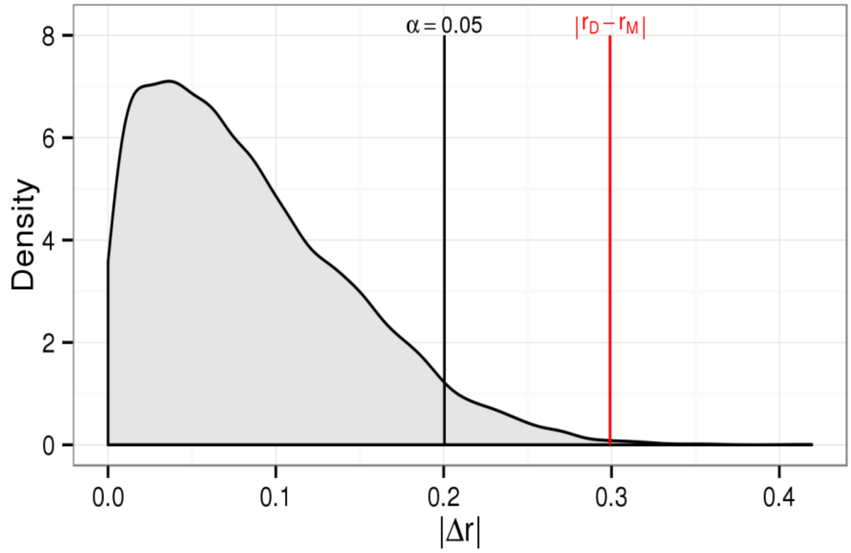

Supplementary Figure 2. Difference of hydrophobicity-to-promiscuity correlation coefficient, r, between drugs (D) and metabolites (M). Pearson correlation coefficients between hydrophobicity (logP) and promiscuity (pocket count) were calculated for both drugs and metabolites separately and subtracted from each other. This was also done for randomly sampled datasets (n=10000), resulting in a significant difference of real and sampled Δr (empirical p-value= 0.0026). Type-I empirical error rate, α, of 5% is indicated by the black vertical line.

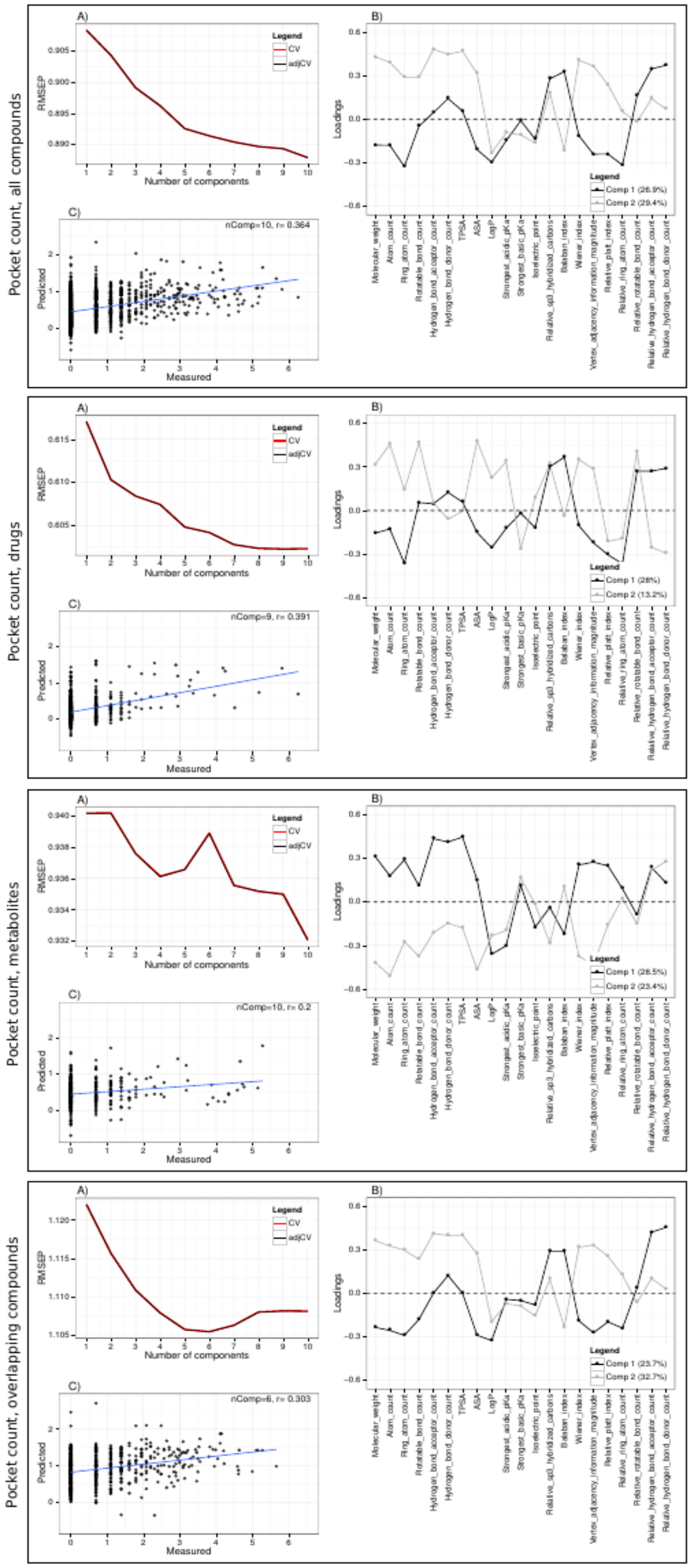

Supplementary Figure 3. Partial least squares regression (PLSR) using physicochemical properties for compound promiscuity (logarithmic pocket count) with regard to compound class. (A) The cross-validated RMSEP (root mean square error of prediction) curves as function of the number of components. (B) The loading plot of the physicochemical properties for the first two components. (C) The measured against predicted values including the number of components (nComp) and correlation coefficient (r).

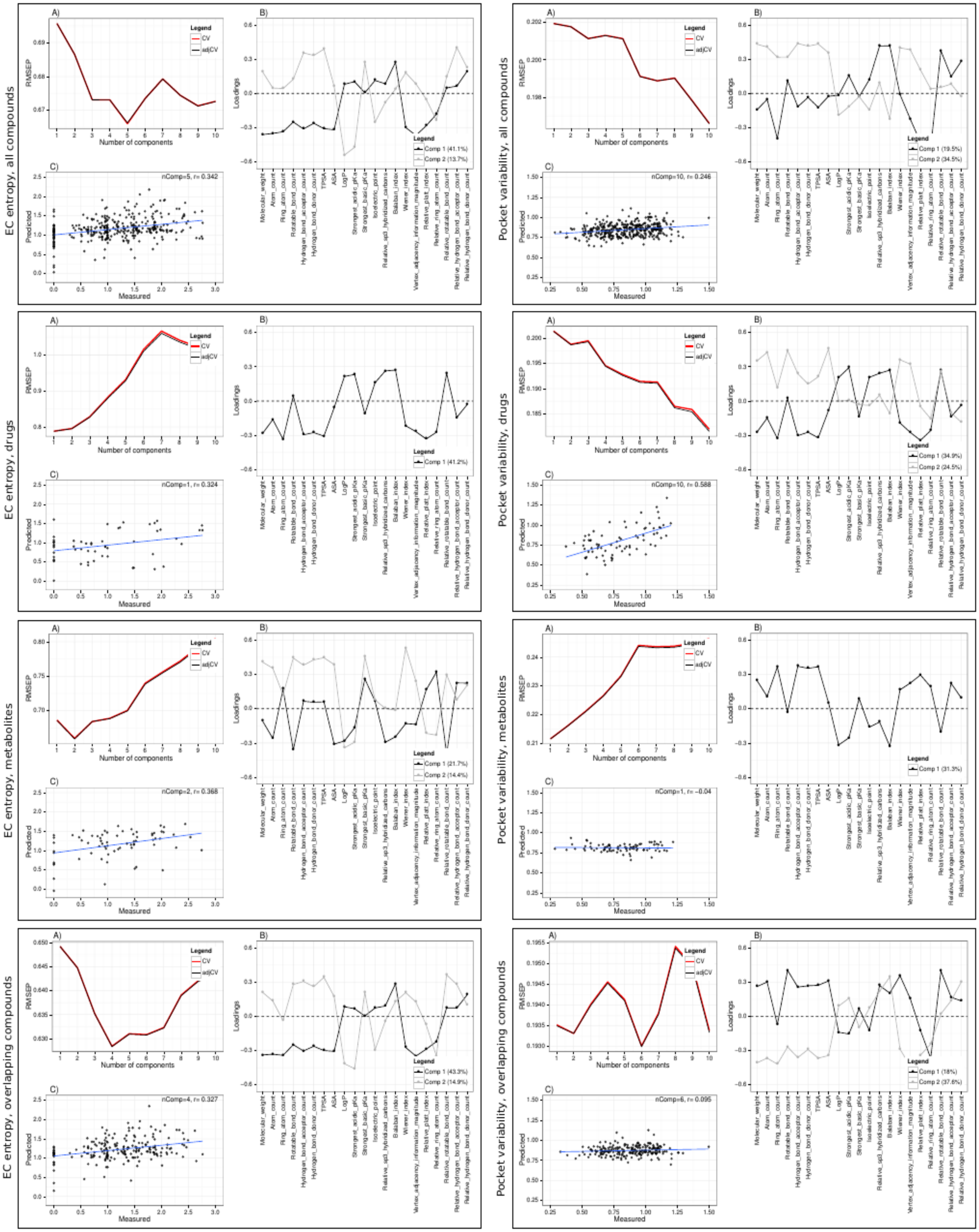

Supplementary Figure 4. Partial least squares regression (PLSR) using physicochemical properties for EC entropy and pocket variability with regard to compound class. (A) The cross-validated RMSEP (root mean square error of prediction) curves as function of the number of components. (B) The loading plot of the physicochemical properties for the first two components. (C) The measured against predicted values including the number of components (nComp) and correlation coefficient (r).

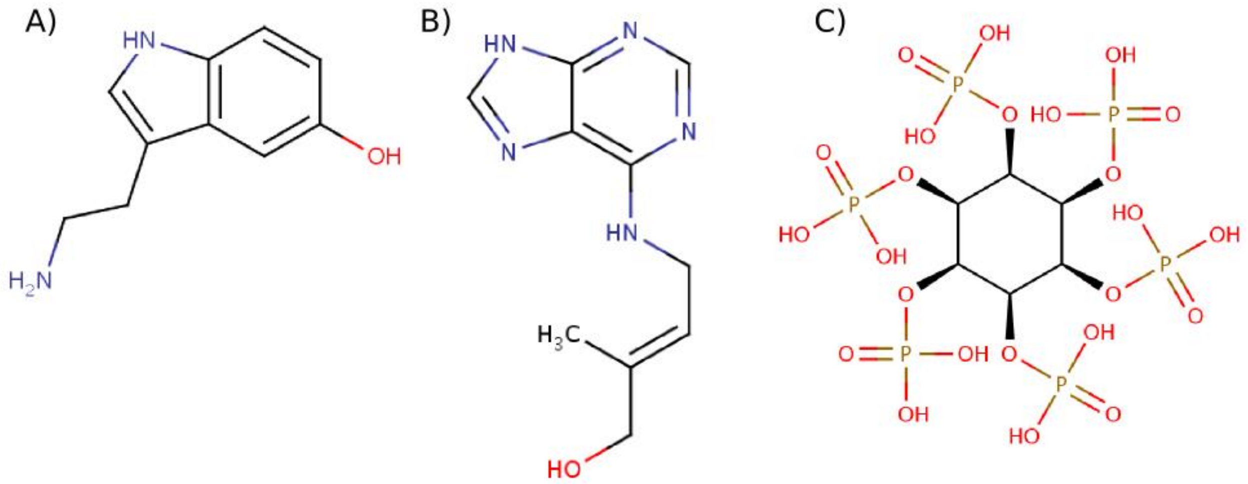

Supplementary Figure 5. Compound structures of metabolites enriched in signal transduction pathways. (A) Serotonin, a common neurotransmitter. (B) Zeatin, a cytokinin and belong to the class of plant growth hormones. (C) Phytate, a phosphorus storage compound in plants with various different biological roles.

Supplementary Table 1. Table of physicochemical properties as calculated with Instant JChem and KNIME including a brief description. (For more details, see following website:

http://onlinelibrarystatic.wiley.com/marvin/help/calculations/calculator-plugins.html).

	Property	Description
Instant JChem	Mol weight	Molecular weight
	Formula	Chemical formula of the molecule according to the Hill system
	atomCount	Number of all atoms in the molecule
	ringAtomCount	Number of ring atoms/ bonds
	rotatableBondCount	Number of rotatable bonds in the molecule. Unsaturated bonds, and single bonds connected to hydrogens or terminal atoms, single bonds of amides, sulphonamides and those connecting two hindered aromatic rings (having at least three ortho substituents) are considered non-rotatable
	acceptorCount	Number of hydrogen bond acceptors
	donorCount	Number of hydrogen bond donors
	TPSA	Topological polar surface area (2D)
	ASA	Solvent accessible surface area of the molecule (3D) (in $Å^2$)
	logP	Calculates the octanol/water partition coefficient; measure of molecular hydrophobicity (Viswanadhan et al.)
	logD	The octanol-water distribution coefficient, logD represents the compounds at any pH value.
	acidicpKa	Strongest acidic pKa; Deprotonated negative ions are considered as deprotonated acidic sites , therefore , "acidic" prefix is used for them.
	basicpKa	Strongest basic pKa; Protonated positive ions are considered as protonated basic sites , therefore , "basic" prefix is used for them.
	isoelectricPoint	Net charge of an ionizable molecule is zero at a certain pH
	fsp3	Number of sp3 hybridized carbons divided by the total carbon count
	Balaban index	Calculates connectivity of the molecule, which is the average distance sum connectivity weighted by bond character (single bond=1, double bond=0.5 etc.)
	Platt index	Sum of the edge degrees of a molecular graph
	Wiener index	The average topological atom distance (half of the sum of all atom distances) in the molecule
KNIME	Vertex adjacency information magnitude	The values is given by 1 + log 2 * m , where m is the number of heavy-heavy bonds. If m is zero, then zero is returned.
	Bond count	Number of heavy bond counts
	No. of heavy atoms	Number of heavy atoms
Instant JChem and KNIME	Relative ring atom count	Ring atom count divided by heavy atom count
	Relative hydrogen bond acceptor count	Hydrogen bond acceptor count divided by heavy atom count
	Relative hydrogen donor acceptor count	Hydrogen donor acceptor count divided by heavy atom count
	Relative rotatable bond count	Rotatable bond count divided by heavy bond count

Supplementary Table 2. Enriched KEGG pathways, processes, and systems in promiscuous or selective metabolite sets. The metabolites are listed according their promiscuity and pathway names, together with their KEGG IDs, corresponding PDB IDs and compound names for all (borderline) significantly enriched pathways in Table 5. Reported categories were found significant at $p_{FDR}<0.2$.

Metabolism			
**Collective**	**KEGG ID**	**PDB ID**	**Compound name**
**Promiscuous: Energy metabolism**	C00024	ACO	Acetyl coenzyme A
	C00008	ADP	ADP; Adenosine 5'-diphosphate
	C00026	AKG	2-Oxoglutarate
	C00002	ATP	ATP; Adenosine 5'-triphosphate
	C00169	CP	Carbamoyl phosphate
	C00234	FFO	10-Formyltetrahydrofolate
	C00037	GLY	Glycine; Aminoacetic acid
	C00565	KEN	Trimethylamine
	C00003	NAD,NAJ	NAD; Nicotinamide adenine dinucleotide
	C00004	NAI	NADH; Reduced nicotinamide adenine dinucleotide
	C00022	PYR	Pyruvate; Pyruvic acid
	C00042	SIN	Succinate; Succinic acid
	C01104	TMO	Trimethylamine N-oxide
**Promiscuous: Metabolism of other amino acids**	C00024	ACO	Acetyl-CoA; Acetyl coenzyme A
	C00026	AKG	2-Oxoglutarate; Oxoglutaric acid
	C00037	GLY	Glycine; Aminoacetic acid; Gly
	C00051	GSH	Glutathione
	C00135	HIS	L-Histidine
	C05335	MSE	L-Selenomethionine
	C00077	ORN	L-Ornithine; (S)-2,5-Diaminovaleric acid
	C00515	ORN	D-Ornithine
	C00864	PAU	Pantothenate; Pantothenic acid
	C00022	PYR	Pyruvate; Pyruvic acid; 2-Oxopropanoate
	C18893	SNG	1-Methylseleno-N-acetyl-D-galactosamine
	C00750	SPM	Spermine
**Promiscuous: Nucleotide metabolism**	C00020	3AM,AMP	AMP; Adenosine 5'-monophosphate
	C01367	3AM,AMP	3'-AMP; 3'-Adenylic acid
	C00559	3D1	Deoxyadenosine; 2'-Deoxyadenosine
	C00147	ADE	Adenine; 6-Aminopurine
	C00008	ADP	ADP; Adenosine 5'-diphosphate
	C03373	AIR	Aminoimidazole ribotide
	C00002	ATP	ATP; Adenosine 5'-triphosphate
	C01260	B4P	P1,P4-Bis(5'-adenosyl)tetraphosphate
	C00169	CP	Carbamoyl phosphate
	C00360	DA	dAMP; 2'-Deoxyadenosine 5'-phosphate
	C00362	DGP	dGMP; 2'-Deoxyguanosine 5'-monophosphate
	C00037	GLY	Glycine; Aminoacetic acid
	C00242	GUN	Guanine
	C00262	HPA	Hypoxanthine
**Selective: Carbohydrate metabolism**	C00100	1VU,SOP	Propionyl coenzyme A
	C01801	2DR	Deoxyribose
	C11544	2M8	2(alpha-D-Mannosyl)-D-glycerate
	C01089	3HL,3HR	(R)-3-Hydroxybutanoate
	C02273	AD0	Digalacturonic acid
	C02713	AMU,MUB	N-Acetylmuramic acid;
	C19972	B6D	2,4-Bis(acetamido)-2,4,6-trideoxy-beta-L-altropyranose
	C20424	B6D	2,4-Diacetamido-2,4,6-trideoxy-D-mannopyranose

	C05993	CA0	Acetyl adenylate
	C06218	CE5,M5S	Cellopentaose
	C06217	CE6	Cellohexaose
	C01159	DG2	2,3-Bisphospho-D-glycerate; DPG
	C00492	DQR,GFG,RAF	Raffinose; Melitose; Melitriose
	C01050	EPZ	UDP-N-acetylmuramate
	C00665	FDP	beta-D-Fructose 2,6-bisphosphate
	C00679	FIX	5-Dehydro-4-deoxy-D-glucarate
	C03921	FIX	2-Dehydro-3-deoxy-D-glucarate
	C00818	GAE,LGT	D-Glucarate
	C00879	GAE,LGT	D-Galactarate
	C01251	HCA	(R)-2-Hydroxybutane-1,2,4-tricarboxylate
	C04573	HP7,MJZ	UDP-N-acetyl-2-amino-2-deoxy-D-glucuronate
	C06240	HP7,MJZ	UDP-N-acetyl-D-mannosaminouronate
	C13952	HP7,MJZ	UDP-N-acetyl-D-galactosaminuronic acid
	C20359	HP7,MJZ	UDP-2-acetamido-3-amino-2,3-dideoxy-alpha-D-glucuronate
	C00691	I1N	2,4,6/3,5-Pentahydroxycyclohexanone
	C20251	I1N	1-Keto-D-chiro-inositol
	C05382	I22	Sedoheptulose 7-phosphate
	C01177	I4C,I4D	Inositol 1-phosphate
	C03546	I4C,I4D	myo-Inositol 4-phosphate
	C04006	I4C,I4D	1D-myo-Inositol 3-phosphate
	C20672	JB3	GDP-4-acetamido-4,6-dideoxy-alpha-D-mannose
	C01235	LB2,M3M	alpha-D-Galactosyl-(1->3)-1D-myo-inositol
	C01620	LTH,THE	Threonate
	C03410	NGE	N-Glycoloyl-neuraminate
	C05401	RGG	3-beta-D-Galactosyl-sn-glycerol
	C01151	RI2	D-Ribose 1,5-bisphosphate
	C00232	SSN	Succinate semialdehyde
	C00149	THE	(S)-Malate
	C03064	THE	3-Dehydro-L-threonate
	C00497	THE	D-Malate
	C04613	UD7	UDP-2-acetamido-4-dehydro-2,6-dideoxyglucose
	C19823	UD7	UDP-2-acetamido-2,6-dideoxy-beta-L-arabino-hexos-4-ulose
	C06118	UNF,UNG	4-(4-Deoxy-alpha-D-gluc-4-enuronosyl)-D-galacturonate
	C05392	UNF,UNG	Oligouronide with 4-deoxy-alpha-L-erythro-hex-4-enopyranuronosyl group
**Selective: Metabolism of terpenoids and polyketides**	C04268	0FX	dTDP-4-amino-4,6-dideoxy-D-glucose
	C11920	0N2	dTDP-L-olivose
	C11910	0N2	dTDP-3-amino-3,4,6-trideoxy-D-glucose
	C18030	0N2	dTDP-2,6-dideoxy-D-kanosamine
	C18092	0N2	dTDP-L-ristosamine
	C01767	0WU	(-)-Carvone
	C11383	0WU	(+)-(S)-Carvone; Carvol
	C00100	1VU,SOP	Propionyl coenzyme A
	C11933	1YF,FNF,QDM	dTDP-D-mycaminose
	C09704	28U	Nerolidol
	C19746	28U	(3R,6E)-Nerolidol
	C06082	A8S	Abscisic acid; ABA
	C03240	DEB	6-Deoxyerythronolide B
	C11922	DWN	dTDP-4-oxo-2,6-dideoxy-D-glucose
	C11927	DWN	dTDP-4-oxo-2,6-dideoxy-L-mannose
	C11928	DWN	dTDP-4-oxo-2,6-dideoxy-D-allose
	C12318	DWN	dTDP-3-amino-2,3,6-trideoxy-D-threo-hexopyranos-4-ulose

	C08592	ECH	Echinenone
	C06653	ERB	Erythromycin B
	C06633	EY5	Erythromycin D
	C11811	H6P	1-Hydroxy-2-methyl-2-butenyl 4-diphosphate
	C20345	IP8	Isopentenyl phosphate
	C00885	ISC	Isochorismic acid
	C11915	JHZ	dTDP-3-methyl-4-oxo-2,6-dideoxy-L-glucose
	C12319	JHZ	dTDP-3-amino-2,3,6-trideoxy-C-methyl-D-erythro-hexopyranos-4-ulose
	C12320	JHZ	dTDP-L-4-oxovancosamine
	C11998	NRB	Narbomycin
	C04146	OTP,VTP,ZTP	all-trans-Octaprenyl diphosphate
	C01107	PMV	(R)-5-Phosphomevalonate
	C11994	PXI	10-Deoxymethymycin
	C12148	SMA	Stigmatellin A
	C15900	SPO	Spheroidene
	C00688	T46,TDO	dTDP-4-dehydro-beta-L-rhamnose
	C11907	T46,TDO	dTDP-4-oxo-6-deoxy-D-glucose
	C11926	T46,TDO	dTDP-4-oxo-6-deoxy-D-allose
	C02029	WA2	Dihydrozeatin
	C01946	ZIO	Oleandomycin; Amimycin
	C16427	ZIR	Isopentenyl adenosine
**Selective: Not assigned**	C11544	2M8	2(alpha-D-Mannosyl)-D-glycerate
	C20571	2WS	alpha-1,5-L-Arabinotriose
	C01089	3HL,3HR	(R)-3-Hydroxybutanoate; (R)-3-Hydroxybutanoic acid
	C11277	5HG	Adefovir; PMEA; 9-(2-Phosphonylmethoxyethyl)adenine
	C06082	A8S	Abscisate; Abscisic acid; ABA
	C06810	AC2	Acyclovir; Aciclovir
	C02273	AD0	Digalacturonate; Digalacturonic acid
	C00483	AEF	Tyramine; 2-(p-Hydroxyphenyl)ethylamine
	C16421	AI2	AI-2; Autoinducer 2
	C02713	AMU,MUB	N-Acetylmuramate; N-Acetylmuramic acid
	C01630	BXP	Xylobiose
	C20570	BXP	alpha-1,5-L-Arabinobiose
	C13051	DN4	Nicotinic acid adenine dinucleotide phosphate; NAADP
	C00492	DQR,GFG,RAF	Raffinose; Melitose
	C00665	FDP	D-Fructose 2,6-bisphosphate
	C20793	GVM	3-Hydroxyoctanoate; 3-Hydroxyoctanoic acid
	C01177	I4C,I4D	Inositol 1-phosphate; myo-Inositol 1-phosphate
	C03546	I4C,I4D	myo-Inositol 4-phosphate
	C04006	I4C,I4D	myo-Inositol 3-phosphate
	C11174	I7P	1-Diphosinositol pentakisphosphate; D-myo-Inositol
	C02165	LTB	Leukotriene B4
	C01598	ML1	Melatonin; N-Acetyl-5-methoxytryptamine
	C01181	NM2	4-Trimethylammoniobutanoate
	C03557	P7I	2-Aminoethylphosphonate
	C00696	PG2	Prostaglandin D2
	C00534	PXM	Pyridoxamine; PM
	C16138	PYL	L-Pyrrolysine; Pyrrolysine
	C06124	S1P	Sphingosine 1-phosphate
	C00149	THE	L-Malate; L-Apple acid; L-Malic acid
	C02477	VIT,VIV	alpha-Tocopherol; Vitamin E
	C05377	VK3	Menadione; Vitamin K3
	C02029	WA2	Dihydrozeatin
	C01946	ZIO	Oleandomycin; Amimycin; Landomycin
**Detailed**	**KEGG ID**	**PDB ID**	**Compound name**
**Selective:**	C04268	0FX	dTDP-4-amino-4,6-dideoxy-D-glucose

**Polyketide sugar unit biosynthesis**	C11920	0N2	dTDP-L-olivose
	C11910	0N2	dTDP-3-amino-3,4,6-trideoxy-D-glucose
	C18030	0N2	dTDP-2,6-dideoxy-D-kanosamine
	C18092	0N2	dTDP-L-ristosamine
	C11933	1YF,FNF,QDM	dTDP-D-mycaminose
	C11922	DWN	dTDP-4-oxo-2,6-dideoxy-D-glucose
	C11927	DWN	dTDP-4-oxo-2,6-dideoxy-L-mannose
	C11928	DWN	dTDP-4-oxo-2,6-dideoxy-D-allose
	C11915	JHZ	dTDP-3-methyl-4-oxo-2,6-dideoxy-L-glucose
	C00688	T46,TDO	dTDP-4-dehydro-beta-L-rhamnose
	C11907	T46,TDO	dTDP-4-oxo-6-deoxy-D-glucose
	C11926	T46,TDO	dTDP-4-oxo-6-deoxy-D-allose
**Environmental Information Processing**			
**Collective**	**KEGG ID**	**PDB ID**	**Compound name**
**Promiscuous: Membrane transport**	C00002	ATP	ATP; Adenosine 5'-triphosphate
	C00114	CHT	Choline; Bilineurine
	C00037	GLY	Glycine; Aminoacetic acid
	C00051	GSH	Glutathione
	C00135	HIS	L-Histidine
	C00077	ORN	L-Ornithine
	C00022	PYR	Pyruvate; Pyruvic acid; 2-Oxopropanoate
**Promiscuous: Signal transduction**	C00020	3AM,AMP	AMP; Adenosine 5'-monophosphate
	C00024	ACO	Acetyl-CoA; Acetyl coenzyme A
	C00008	ADP	ADP; Adenosine 5'-diphosphate
	C00026	AKG	2-Oxoglutarate; Oxoglutaric acid
	C00002	ATP	ATP; Adenosine 5'-triphosphate
	C16463	C2E	3',5'-Cyclic diGMP
	C01204	IHP	Phytic acid; Phytate
	C09731	KDH	Epigallocatechin 3-gallate
	C00003	NAD,NAJ	NAD; Nicotinamide adenine dinucleotide
	C00004	NAI	NADH; Reduced nicotinamide adenine dinucleotide
	C00022	PYR	Pyruvate; Pyruvic acid
	C00042	SIN	Succinate; Succinic acid
	C00780	SRO	Serotonin
	C01104	TMO	Trimethylamine N-oxide
	C00371	ZEA	Zeatin
**Promiscuous: Signaling molecules and interaction**	C00008	ADP	ADP; Adenosine 5'-diphosphate
	C00002	ATP	ATP; Adenosine 5'-triphosphate
	C00037	GLY	Glycine; Aminoacetic acid; Gly
	C00388	HSM	Histamine; 1H-Imidazole-4-ethanamine
	C00780	SRO	Serotonin; 3-(2-Aminoethyl)-1H-indol-5-ol
	C00008	ADP	ADP; Adenosine 5'-diphosphate
	C00002	ATP	ATP; Adenosine 5'-triphosphate
	C00037	GLY	Glycine; Aminoacetic acid
	C00388	HSM	Histamine; 1H-Imidazole-4-ethanamine
	C00780	SRO	Serotonin; 3-(2-Aminoethyl)-1H-indol-5-ol
**Detailed**	**KEGG ID**	**PDB ID**	**Compound name**
**Promiscuous: AMPK signaling pathway**	C00020	3AM,AMP	AMP; Adenosine 5'-monophosphate
	C00024	ACO	Acetyl coenzyme A
	C00008	ADP	ADP; Adenosine 5'-diphosphate
	C09731	KDH	Epigallocatechin 3-gallate
	C00003	NAD,NAJ	NAD; Nicotinamide adenine dinucleotide
	C00022	PYR	Pyruvate; Pyruvic acid
**Promiscuous: HIF-1 signaling**	C00024	ACO	Acetyl coenzyme A
	C00026	AKG	2-Oxoglutarate; Oxoglutaric acid

pathway	C00002	ATP	ATP; Adenosine 5'-triphosphate
	C00022	PYR	Pyruvate; Pyruvic acid
**Organismal Systems**			
**Collective**	**KEGG ID**	**PDB ID**	**Compound name**
**Promiscuous: Digestive system**	C00024	ACO	Acetyl coenzyme A
	C00026	AKG	2-Oxoglutarate; Oxoglutaric acid
	C00114	CHT	Choline; Bilineurine
	C00010	COA	CoA; Coenzyme A
	C00037	GLY	Glycine; Aminoacetic acid
	C00051	GSH	Glutathione
	C00135	HIS	Histidine
	C00388	HSM	Histamine
	C00463	IND	Indole; 2,3-Benzopyrrole
	C00003	NAD,NAJ	NAD; Nicotinamide adenine dinucleotide
	C00864	PAU	Pantothenic acid; vitamin B5
	C00018	PLP	Pyridoxal phosphate; vitamin B6 phosphate
	C00250	PXL	Pyridoxal; vitamin B6
	C00255	RBF	Riboflavin; Vitamin B2
	C00376	RET	Retinal; Vitamin A aldehyde
	C00473	RTL	Retinol; Vitamin A
	C00750	SPM	Spermine
	C00780	SRO	Serotonin
	C00078	TRP	Tryptophan
	C00378	VIB	Thiamine; Vitamin B1
**Promiscuous: Endocrine system**	C00002	ATP	ATP; Adenosine 5'-triphosphate
	C00051	GSH	Glutathione
	C00022	PYR	Pyruvate; Pyruvic acid; 2-Oxopropanoate
**Promiscuous: Nervous system**	C00024	ACO	Acetyl coenzyme A
	C00026	AKG	2-Oxoglutarate; Oxoglutaric acid
	C00002	ATP	ATP; Adenosine 5'-triphosphate
	C00114	CHT	Choline; Bilineurine
	C00010	COA	CoA; Coenzyme A
	C00037	GLY	Glycine; Aminoacetic acid
	C00388	HSM	Histamine
	C00042	SIN	Succinic acid
	C00780	SRO	Serotonin
	C00078	TRP	Tryptophan
**Promiscuous: Sensory system**	C00002	ATP	ATP; Adenosine 5'-triphosphate
	C00388	HSM	Histamine
	C00376	RET	Retinal; Vitamin A aldehyde
	C02110	RET	11-cis-Retinal; 11-cis-Vitamin A aldehyde
	C00780	SRO	Serotonin
**Detailed**	**KEGG ID**	**PDB ID**	**Compound name**
**Promiscuous: Vitamin digestion and absorption**	C00010	COA	CoA; Coenzyme A
	C00003	NAD,NAJ	NAD; Nicotinamide adenine dinucleotide
	C00864	PAU	Pantothenic acid; vitamin B5
	C00018	PLP	Pyridoxal phosphate; vitamin B6 phosphate
	C00250	PXL	Pyridoxal; vitamin B6
	C00255	RBF	Riboflavin; Vitamin B2
	C00376	RET	Retinal; Vitamin A aldehyde
	C00473	RTL	Retinol; Vitamin A
	C00378	VIB	Thiamine; Vitamin B1
**Promiscuous: Bile secretion**	C00024	ACO	Acetyl-CoA; Acetyl coenzyme A
	C00026	AKG	2-Oxoglutarate; Oxoglutaric acid
	C00114	CHT	Choline; Bilineurine

	C00051	GSH	Glutathione
	C00750	SPM	Spermine
	C00780	SRO	Serotonin
**Promiscuous: Protein digestion and absorption**	C00037	GLY	Glycine; Aminoacetic acid; Gly
	C00135	HIS	L-Histidine
	C00388	HSM	Histamine
	C00463	IND	Indole; 2,3-Benzopyrrole
	C00078	TRP	L-Tryptophan; Tryptophan
	C00037	GLY	Glycine; Aminoacetic acid; Gly
	C00135	HIS	L-Histidine
**Promiscuous: Synaptic vesicle cycle**	C00002	ATP	ATP; Adenosine 5'-triphosphate
	C00037	GLY	Glycine; Aminoacetic acid; Gly
	C00388	HSM	Histamine
	C00780	SRO	Serotonin

Supplementary Table 3. Compound class specific binary prediction of compounds as promiscuous or specific using SVMs. Listed are the fractions of wrong classifications in cross-validation on equally sized datasets (114 promiscuous/selective drugs and 129 promiscuous/selective metabolites). The larger selective datasets were sampled 10 times and averaged. A range of different kernels including a linear and several non-linear functions (RBF- radial basis function) was applied. Training error relates to the "self-test", while a 5-fold cross-validation was performed to estimate the true prediction performance on new datasets. Smallest classification errors in each set are underlined.

	**Drugs**		**Metabolites**	
**Kernel**	**Training error**	**Cross-validation error**	**Training error**	**Cross-validation error**
**Linear**	0.2254	0.2688	0.3519	0.4558
**Polynomial**	0.2531	0.3075	0.3407	<u>0.4430</u>
**RBF**	0.1904	0.2701	0.3023	0.4798
**Laplacian**	0.2311	<u>0.2609</u>	0.3349	0.5261
**Anova RBF**	<u>0.1320</u>	0.3214	<u>0.2120</u>	0.4511
